# Pathogen distribution and antimicrobial resistance among neonatal bloodstream infections in Southeast Asia: results from NeoSEAP, a multicentre retrospective study

**DOI:** 10.1016/j.lanwpc.2025.101617

**Published:** 2025-09-09

**Authors:** Benjamin F.R. Dickson, Michelle Harrison, Maria Esterlita T. Villanueva-Uy, Nina Dwi Putri, Riyadi Adrizain, Leny Kartina, Gayana P.S. Gunaratna, Hoang T. Tran, Nguyen X. Huong, Siew M. Fong, Erena S. Kasahara, Distyayu Sukarja, Tetty Yuniati, Martono Utomo, Nambage S. Chandrasiri, Chau H.M. Le, Nguyen T.K. Trinh, Ng Boon Hong, Hoang N.T. Thuy, Tran T.C. Tu, Le T. Hong, Jannah Baker, Mark Jones, Thomas L. Snelling, Mike Sharland, Phoebe C.M. Williams, NeoSEAP Consortium

**Affiliations:** a School of Public Health, Faculty of Medicine & Health, The University of Sydney, Sydney, Australia; b Sydney Institute for Infectious Diseases, The University of Sydney, Sydney, Australia; c Institute of Child Health and Human Development, National Institutes of Health, Manila, Philippines; d Philippine General Hospital, Manila, Philippines; e Cipto Mangunkusumo Hospital, Jakarta, Indonesia; f Universitas Indonesia, Jakarta, Indonesia; g Hasan Sadikin General Hospital, Bandung, Indonesia; h Department of Child Health, Faculty of Medicine, Universitas Padjadjaran, Bandung, Indonesia; i Soetomo Hospital, Surabaya, Indonesia; j University of Airlangga, Surabaya, Indonesia; k Faculty of Medicine, University of Kelaniya, Colombo, Sri Lanka; l Colombo South Teaching Hospital, Colombo, Sri Lanka; m Department of Pediatrics, School of Medicine and Pharmacy, The University of Da Nang, Da Nang, Vietnam; n Da Nang University of Medical Technology and Pharmacy, Da Nang, Vietnam; o Phan Chau Trinh University, Quang Nam, Vietnam; p Sabah Women's and Children's Hospital, Sabah, Malaysia; q Da Nang Tam Tri General Hospital, Da Nang, Vietnam; r Nha Trang Tam Tri General Hospital, Nha Trang, Vietnam; s Dong Thap Tam Tri General Hospital, Dong Thap, Vietnam; t St George's University, London, United Kingdom; u Department of Infectious Diseases, Sydney Children's Hospital Network, Australia; v Da Nang Hospital for Women and Children, Da Nang, Vietnam

**Keywords:** Neonatal sepsis, Antimicrobial resistance, Gram-negative bacteria, Southeast Asia, Global health

## Abstract

**Background:**

Progress in reducing morbidity and mortality due to neonatal sepsis has slowed in recent decades and is threatened by the global rise of antimicrobial resistance. The populous Southeast Asian region has a high burden of both neonatal sepsis and antimicrobial resistance (AMR). Despite this, there remains a lack of robust data on the epidemiology of neonatal sepsis and the prevalence of AMR within the region.

**Methods:**

We evaluated positive blood cultures and susceptibility profiles responsible for neonatal sepsis across 10 clinical sites in five countries in South and Southeast Asia (Sri Lanka, Indonesia, The Philippines, Malaysia, and Vietnam). Retrospective data on all blood cultures collected from neonates over two years (1st January 2019–31st December 2020) were extracted from laboratory records. Data were also collected on the availability and implementation of infection prevention and control resources, and antimicrobial prescribing practices. Pooled estimates across sites and pathogens were generated, with adjustment for clustering.

**Findings:**

Of 14,804 blood cultures collected over the study period, a total of 2131 positive isolates (including 1483 significant pathogens) were identified. Gram-negative bacteria predominated as causative of neonatal sepsis (78·4%; 1163/1483) with *Klebsiella* spp. (408/1483; 27·5%) and *Acinetobacter* spp. (261/1483; 17·6%) most frequently isolated overall. Adjusted pooled non-susceptibility for *Klebsiella* spp. was 86·7% (95% CI 54·0–98·5) for third-generation cephalosporins (ceftriaxone and/or cefotaxime; 3GC) and 17·1% (95% CI 8·1–24·7) for carbapenems; while non-susceptibility for *Escherichia coli* was 46·4% (95% CI 20·0–72·0) for 3GC and 15·4% (95% CI 2·7–31·0) for carbapenems. Carbapenem non-susceptibility for *Acinetobacter* spp. was 76·5% (95% CI 59·4–84·5). Gram-positive bacteria accounted for 13·2% (196/1483) of pathogens causative of neonatal sepsis, whilst *Candida* spp. accounted for 8·3% (123/1483) of culture-positive sepsis episodes.

**Interpretation:**

Neonatal sepsis in tertiary hospitals in Southeast Asia is predominantly caused by gram-negative bacteria, with high rates of non-susceptibility to commonly prescribed antibiotics.

**Funding:**

This study was supported by an 10.13039/501100000925Australian National Health and Medical Research Council (NHMRC) grant. The NHMRC was not involved in the design or conduct of the research.

**Research in context**

**Evidence before this study**

Increasing evidence suggests that neonatal sepsis in resource-constrained health settings is predominantly caused by gram-negative bacteria, with a high burden of antimicrobial resistance (AMR) to empirical treatment regimens recommended by the World Health Organization (WHO). However, there remains a lack of robust and granular data on the epidemiology of neonatal sepsis in one of the world's most populous regions, Southeast Asia.

**Added value of this study**

We systematically evaluated the causative pathogens and susceptibility profiles of all neonatal blood cultures across 10 clinical sites in five countries in South and Southeast Asia over a 24-month period, revealing alarming levels of non-susceptibility to commonly recommended and prescribed empirical antibiotic regimens for neonatal sepsis.

**Implications of all the available evidence**

Our results reveal high rates of AMR in neonatal sepsis across Southeast Asia, highlighting a need for updated empirical therapy regimens with improved coverage against contemporary causes of neonatal infections, to reduce the morbidity and mortality associated with neonatal sepsis. There is also a need for prioritised development and licencing of novel antimicrobial agents to treat multidrug-resistant infections in neonates, alongside ensuring their accessibility in regions where the burden of neonatal sepsis is greatest.

## Introduction

Significant progress has been made in reducing child mortality in recent decades.^1^ Despite this, there have been only modest improvements in neonatal mortality rates.^1^, ^2^, ^3^ Neonatal sepsis remains a major cause of neonatal morbidity and mortality globally, responsible for an estimated half a million deaths in infants each year.^2^^,^^4^ Many survivors face long-term sequelae, including higher post-discharge mortality rates, and cognitive and physical disabilities.^5^ The burden of neonatal sepsis disproportionally affects low- and middle-income countries (LMICs), where 85% of neonatal sepsis-related deaths occur.^5^ This burden is now further compounded by the rise of antimicrobial resistance (AMR), which threatens to halt, or even reverse, recent global health gains.^6^

Antimicrobial resistance most significantly affects resource-constrained clinical settings, where frequently overwhelmed healthcare systems are less able to confront this emerging challenge.^6^, ^7^, ^8^ Neonates are particularly vulnerable to the rise of AMR.^7^^,^^8^ The rapid progression and non-specific presentation of neonatal sepsis drives high rates of empirical antimicrobial use, which promotes selection pressure in hospital settings.^9^, ^10^, ^11^ Simultaneously, premature infants experience long hospital admissions, increasing the probability of multidrug-resistant organism (MRO) colonisation and subsequent infection.^7^ This risk is most significant in LMICs, where sepsis is predominantly caused by gram-negative bacteria, and crowded health systems foster horizontal transmission of MROs.^12^ Emerging data from Africa and Asia have demonstrated concerning levels of non-susceptibility to empirical first-line (ampicillin/benzylpenicillin plus gentamicin) and alternative (third-generation cephalosporins) treatment regimens for neonatal sepsis recommended by the World Health Organization (WHO); subsequently, it is now estimated that one-third of neonatal sepsis deaths are directly attributable to AMR.^13^, ^14^, ^15^, ^16^, ^17^, ^18^, ^19^, ^20^

The populous area encompassing the WHO-defined Southeast Asian and Western Pacific Regions collectively account for both the highest absolute number of neonatal sepsis cases and the total annual deaths attributable to AMR.^21^^,^^22^ Recent systematic reviews suggest that treatment coverage provided by WHO-recommended therapies for neonatal sepsis in the region are below 50%.^20^^,^^23^^,^^24^ Concurrently, these reviews highlighted the paucity of robust, data on the epidemiology of neonatal sepsis and AMR in this region, with overrepresentation from larger countries (such as India and China) frequently biasing pooled estimates.^23^ Without representative granular data from all Southeast Asian countries, data will not accurately reflect the contemporaneous causes of neonatal sepsis or the AMR burden in the region. These data are required to ensure the current simplified global empirical treatment guidelines remain appropriate across regions in the era of rising AMR.

Non-representative guidelines are particularly problematic for treating neonatal sepsis, as ‘culture-negative’ neonatal sepsis is a frequently encountered clinical challenge due to difficulties in attaining blood volumes that facilitate adequate sensitivity of culture results, compounded by restricted laboratory capacity in many resource-constrained healthcare settings, where the neonatal sepsis burden is greatest.^9^ Treatment decisions are therefore heavily reliant on empirically recommended regimens, with many guidance policies—such as the WHO Pocketbook—not updated to reflect the contemporaneous epidemiology of AMR within neonatal sepsis.^19^^,^^25^ In the absence of guidance aligned to the local epidemiology of neonatal sepsis, and in the face of growing rates of non-susceptibility to empirical treatment recommendations, prescribing of broad-spectrum empirical antibiotics increases, further driving selection pressure and increasing the regional and global burden of AMR.^9^^,^^11^

This study, undertaken by the Neonatal sepsis in Southeast Asia and the Pacific (NeoSEAP) collaboration, sought to address these research gaps by establishing granular data on the epidemiology of neonatal sepsis and burden of AMR in the populous region of Southeast Asia. Our primary aim is to inform the appropriateness of currently-recommended antibiotic regimens against contemporary causes of neonatal sepsis.^19^ To provide context to these microbiological data, we also assessed the availability of local healthcare resources, the implementation of infection prevention and control (IPC) strategies, and antimicrobial prescribing practices at each clinical site. These data will inform national, regional, and global AMR surveillance programs by providing neonatal AMR estimates,^21^ whilst also contributing to global efforts to evaluate updated empirical treatment regimens,^26^ and to enable prioritised development of novel antimicrobials effective against the contemporary causes of neonatal sepsis.^27^

## Methods

### Study design

This multicentre, observational study was conducted in 10 clinical sites in five South and Southeast Asian countries (Sri Lanka, Indonesia, The Philippines, Vietnam, and Malaysia). To enable comparability with other neonatal sepsis observational studies,^15^ we utilised similar methodology and incorporated three data components: 1) a survey of available healthcare resources and IPC strategies; 2) a retrospective audit of blood cultures collected from neonates (aged 0–28 days inclusive) over a 24-month period; and 3) an antimicrobial prescribing point prevalence survey (PPS) to understand local antibiotic use.

### Site selection

Countries with identified gaps in published data pertaining to the epidemiology of neonatal sepsis and AMR epidemiology were included. Convenience sampling was used to select comparable healthcare settings across each country, consistent with prior neonatal observational studies.^9^^,^^15^ Microbiology and resource requirements needed to participate in the study precluded random sampling.

### Data collection

Data were systemically collected using the REDCap (www.redcap.org) electronic platform.

### Infection prevention and control resources

Hospital resources and IPC measures were assessed using a pre-coded questionnaire at a single timepoint between December 2021 and April 2023 (Supplemental Table S1). Local clinical and research staff collected data from hospital records with assistance from hospital IPC teams. Collected data was verified by direct observation during coordinating investigator visits at the study sites.

### Microbiological analysis

Microbiological data from all blood cultures collected from neonates between 1st January 2019 and 31st December 2020 were systematically extracted from electronic and/or paper laboratory records at each site. Pre-defined contaminants (including coagulase-negative Staphylococci [CoNS], *Corynebacterium* spp., *Fusobacterium* spp., *Micrococcus* spp., *Propionibacterium* spp., and *Bacillus* spp.) were excluded from the analysis.^25^^,^^28^ Susceptibility data for a pre-defined list of pathogenic bacteria and fungi in accordance with published literature (Supplemental Table S2) were collected.^15^ Positive cultures from the same patient with the same organism collected within four weeks were considered to occur as part of the same infectious episode, and were removed as duplicates.

Blood cultures were collected by local staff for suspicion of infection and processed with the BacTec FX System (Becton Dickinson, Sparks USA) and/or BacT/ALERT 3D (bioMerieux, Inc. Durham USA) automated systems. Positive cultures underwent identification and antimicrobial susceptibility testing using VITEK-2 (BioMerieux, Inc. Durham, USA) or Phoenix (Becton Dickinson, Sparks USA) automated analysers, alongside conventional methods. At the Sri Lankan site, Enterobacterales identification was limited to lactose-fermentation status due to resource constraints. Antimicrobial susceptibility results were interpreted at each site in accordance with breakpoints established by Clinical and Laboratory Standards Institute.^29^ Intermediate results were classified as resistant.

### Data collection: antibiotic prescribing

An antibiotic PPS was conducted to assess antibiotic prescribing for admitted infants at 8am on a pre-defined date (between December 2022 and January 2023). Demographic, clinical and treatment data were extracted from hospital records by clinical and research staff. The results of the PPS are published separately.^30^

### Statistical analysis

Data analysis was conducted using R (version 4·2·1; R Foundation for Statistical Computing, Austria) within the RStudio environment (version 12·0; RStudio, USA). The pathogen distribution and their antimicrobial non-susceptibility were calculated for each site. Estimates of pathogen non-susceptibility against key antimicrobials, pooled across sites, were generated with 95% confidence intervals, before and after adjustment for clustering at the site level. Separately, estimates of non-susceptibility for combined Enterobacterales against key antimicrobials were generated with adjustment for clustering by species. Estimates were produced using a binomial generalised linear model that included applicable random effects via the *glmer* function in the *lme4* package. For pathogen-antibiotic combinations with sparse data, bootstrapping with 1000 iterations was employed via the *bootMer* function. For combinations where no resistance was detected, unadjusted one-sided confidence intervals were calculated using the Wilson-score interval method.

### Ethical approval

This was an observational study with no patient identifiers collected, and no interventions performed. A waiver of consent was granted at most sites, with formal ethical approval obtained where locally required (Universitas Indonesia, Jakarta; Dr. Soetomo Hospital, Surabaya; RSUP Dr. Hasan Sadikin, Bandung; Colombo South Teaching Hospital, Colombo and University of Philippines, Manila).

This paper is reported in accordance with the Strengthening the Reporting of Observational Studies in Epidemiology (STROBE) and for STROBE for Newborn Infections (STROBE-NI) recommendations.^31^^,^^32^

### Role of the funding source

This study was supported by an Australian National Health and Medical Research Council (NHMRC) grant. The NHMRC was not involved in the design or conduct of the research.

## Results

### Site characteristics

**Fig. 1:**
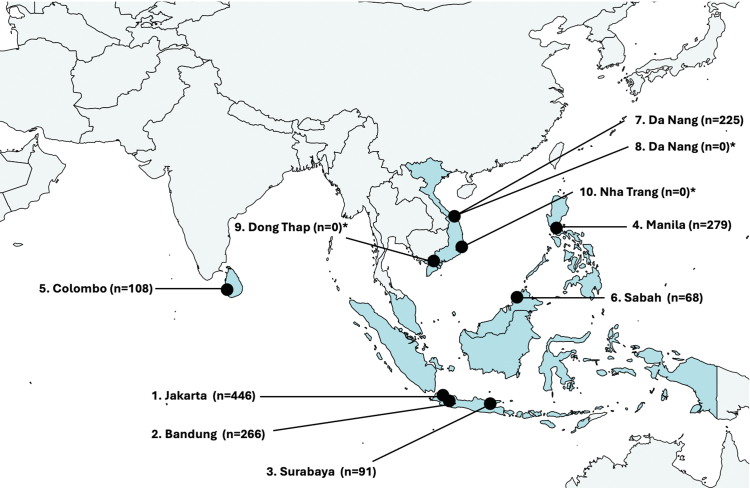
**Map of included countries and study sites.** The number preceding each city denotes site number. The number in parentheses denotes number of positive blood cultures at each site for the period of 2019–2020 inclusive. ∗Private hospital.

Fig. 1 and Table 1 summarise study sites characteristics. Seven sites were public tertiary hospitals, while three were private referral hospitals, all located in Vietnam. All but one rural Vietnamese site were located in urban areas. All hospitals had on-site delivery facilities, with a median of 1595 births per year (interquartile range (IQR): 1308–4918), while also serving as referral centres for clinically-unstable infants. The median bed capacity was 63 beds (IQR: 24–92) for special care nurseries and intensive care units, and 35 beds (IQR: 25–48) for postnatal wards. Hospitals reported a median of 1169 (IQR: 247–1895) annual postnatal admissions, with a median proportion of premature (<37 weeks' gestation) admitted infants of 42% (IQR: 11–52%). All sites total parenteral nutrition access, most (90%) had incubators and invasive ventilation (80%), while 40% had established human donor milk programs.

**Table 1:** Characteristics of NeoSEAP collaboration sites.

Country	Indonesia			Philippines	Sri Lanka	Malaysia	Vietnam			
Site	1	2	3	4	5	6	7	8	9	10
Ownership & type	Public tertiary	Public tertiary	Public tertiary	Public tertiary	Public tertiary	Public tertiary	Public tertiary	Private referral	Private referral	Private referral
Location	Urban	Urban	Urban	Urban	Urban	Urban	Urban	Urban	Rural	Urban
Beds (n)										
Postnatal ward	2	24	28	48	30	104	107	25	40	40
Special care nursery	17	24	40	30	6	58	12	10^a^	54^a^	6
HDU	19	44	32	0	10	24	111			0
ICU	30	14	20	30	8	18	49			0
Total	68	106	120	108	54	204	279	35	94	46
Neonatal staffing (Total FTE^b^)										
Nurses	116	64	53	42	25	162	61	3	0^c^	12
Trainee doctor	14	7	8	24	12	25	14	1	0^c^	4
Consultant	6	3	4	7	2	1	5	1	0^c^	1
Deliveries										
Deliveries on-site	Yes	Yes	Yes	Yes	Yes	Yes	Yes	Yes	Yes	Yes
Birth per year (n)	1508	1681	853	3365	4918	14,052	10,159	492	1308	1339
Admissions (n)										
Total	1433	1895	1490	904	398	6081	2448	52	215	247
Premature	823	797	715	864	206	986	1023	1	12	28
Services										
Neonatal surgery	Yes	Yes	Yes	Yes	No	Yes	Yes	Yes	No	No
Incubators (n)	42	40	43	27	16	29	17	1	0	1
Non-invasive ventilation	Yes	Yes	Yes	Yes	Yes	Yes	Yes	Yes	No	Yes
Invasive ventilation	Yes	Yes	Yes	Yes	Yes	Yes	Yes	Yes	No	No
Inotropes	Yes	Yes	Yes	Yes	Yes	Yes	Yes	No	Yes	Yes
TPN	Yes	Yes	Yes	Yes	Yes	Yes	Yes	Yes	Yes	Yes
Donor-milk	Yes	No	No	Yes	No	No	Yes	No	No	Yes

TPN: Total parenteral nutrition; HDU: High dependency unit; ICU: intensive care unit.

a Neonatal beds located paediatric ward. For site 8, ten dedicated neonatal beds are available. For site 9 the number reflects total mixed paediatric/neonatal bed capacity.

b Full time equivalent (FTE).

c No dedicated neonatal staff available, paediatric staff care for neonates and children.

### IPC strategies

**Fig. 2:**
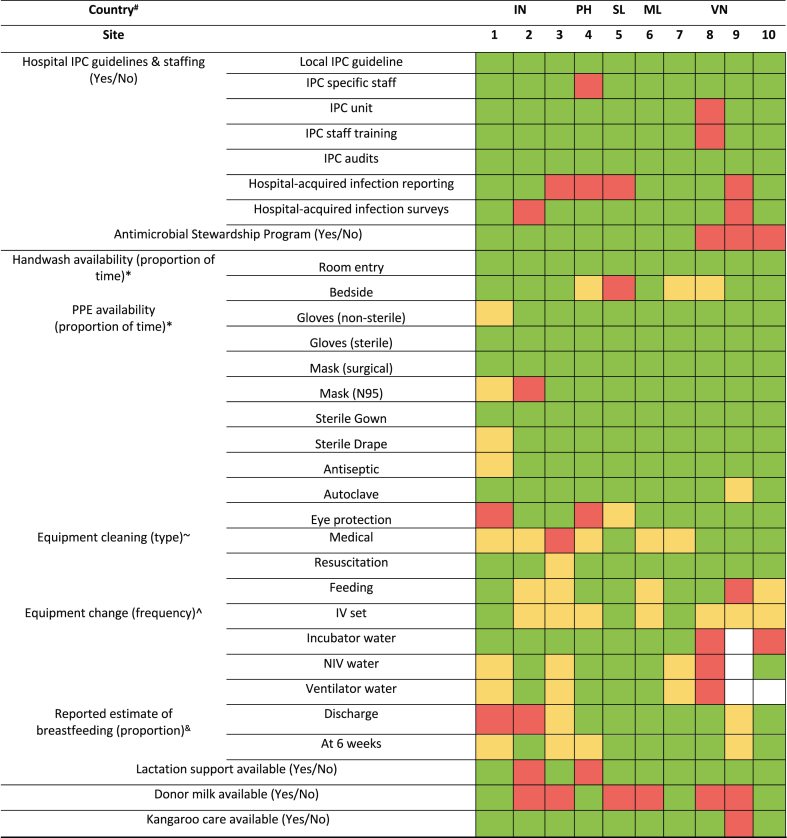
**Heatmap of resource availability and infection prevention & control (IPC) strategies by site.** ∗Criteria: frequency of time: Green (100%), Yellow (50–100%), Red (<50%). ∼Criteria: Green (Sterilisation), Yellow (Disinfection), Red (Soap & water). ˆCriteria: Green (Daily), Yellow (2–3 times per week), Red (Less than 2–3 per week), White (Not applicable). ^&^Criteria: Green (76–100%), Yellow (50–75%), Red (<50%). Data based on available hospital records. ^#^Country: Indonesia (IN), Philippines (PH), Sri Lanka (SL), Malaysia (ML), Vietnam (VN). IPC: Infection, Prevention and Control; IV: Intravenous; NIV: Non-invasive Ventilation.

Fig. 2 presents a heatmap of IPC resources and strategies across sites. All had established IPC guidelines, and most (90%) employed dedicated IPC staff. Antiseptic hand rub was ‘always available’ in 60% of sites, while gloves were available in 90%. Median estimated proportion of infants breastfeeding at discharge was 80% (IQR: 70–92%). Kangaroo care was routinely practiced during the perinatal period at most sites (9/10, 90%); however, its use was reported to be less common in the postnatal period due to space constraints.

### Blood culture collection and empirical treatment regimes

Table 2 outlines the blood culture collection practices and empirical antibiotic regimens across sites. Seven sites routinely collected blood cultures before initiating antibiotics. However, in some sites, blood culture collection was intermittently hindered by limited availability of laboratory consumables and scientists to process samples. A blood culture was considered ‘negative’ at 48 h by most sites (range: 48 h–five days).

**Table 2:** Empirical antimicrobial recommendations and blood culture practices by site.

EOS: Early onset neonatal sepsis; LOS: late onset neonatal sepsis (onset > 72 h of life).

^a^Colour refers AWaRe classification: Access (green), Watch (yellow), Reserve (red; none identified). All antibiotic doses are intravenous. OD (Once daily), BD (Twice daily), TDS (Three times daily), QID (Four times daily), AMP (Ampicillin), GEN (Gentamicin), AMK (Amikacin), MEM (Meropenem), CAZ (Ceftazidime), CTX (Cefotaxime or Ceftriaxone).

^b^BD < 7 days old, TDS > 7 days old.

^c^Nystatin dose: 100,000 international units PO TDS; Fluconazole dose 3 mg/kg (orally) twice per week.

^d^Criteria: <1500 g.

^e^Criteria: Weight < 1500 g, or gestational age < 32 weeks.

^f^Criteria: Weight ≤ 1500 g plus broad spectrum Abx, or weight > 1500 g and nil-by-mouth for >5 days.

^g^Criteria: Clinician discretion.

Local empirical treatment guidelines for neonatal sepsis and meningitis were informed by multiple sources, including national and local guidelines, as well as recommendations from WHO, and United Kingdom National Institute for Clinical Evidence (NICE) (Table 2). Most national (90%, 9/10) and nearly half of local (44%, 4/9) guidelines followed were published before 2020.

For early-onset sepsis (defined as sepsis occurring within the first 72 h of life), the predominant empirical antibiotic regimen was ampicillin plus gentamicin (70%). By contrast, regimens for neonatal meningitis and late-onset sepsis (defined as sepsis occurring after 72 h of life) were more varied (Table 2). Late-onset sepsis regimes frequently included an aminoglycoside plus a penicillin-based antibiotic, and/or non-anti-pseudomonal third-generation cephalosporin (ceftriaxone and/or cefotaxime; 3GC). Antibiotics classified in the WHO ‘Watch’ category were included in late-onset regimens at six sites (60%), and in meningitis regimes at eight sites (80%). Four sites (40%) prescribed antifungal prophylaxis (nystatin or fluconazole) for premature infants at high risk of invasive fungal infections. Seven (70%) sites had established antimicrobial stewardship programs.

### Blood cultures

Of 14,804 blood cultures collected over the study period, there were a total of 2131 positive isolates including 1483 significant pathogens identified (Table 3, Supplemental Figure S1). The median blood culture positivity rate (from sites with available denominator data) was 7·4% (IQR: 5·3–19·5%).

**Table 3:** Blood culture denominators by site.

Country	Indonesia			Philippines	Sri Lanka	Malaysia	Vietnam				Total
Site	1	2	3	4	5	6	7	8	9	10	
Blood cultures analysed	2059	1649	1731	3831	–	2525	3004	0	0	5	14,804
Positive isolates											
Total (including contaminants)	614	322	179	525	124	94	273	0	0	0	2131
Total (excluding contaminants)	446	266	91	279	108	68	225	0	0	0	1483
Total captured^a^	438	249	80	279	103	61	65	0	0	0	1275
Gram-positive	18	26	12	14	26	25	75	0	0	0	196
Gram-negative	406	218	68	231	76	43	121	0	0	0	1163
Fungal	22	22	11	34	6	0	29	0	0	0	124
Total with AST data^b^	407	208	76	278	99	61	45	0	0	0	1174

a Captured organisms refers to those captured in the study from a pre-specified list, other pathogens were grouped as ‘other’.

b AST (Antimicrobial susceptibility testing).

### Pathogen distribution

**Fig. 3:**
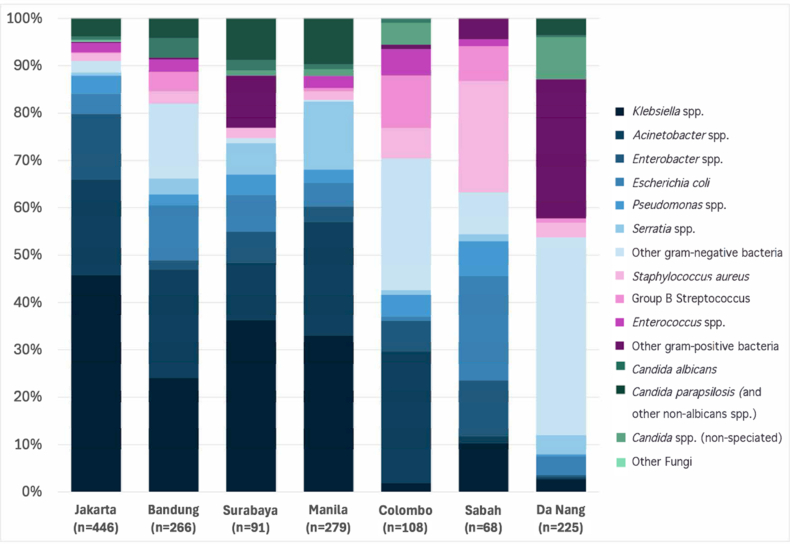
**Distribution of culture-positive pathogens isolated across all sites (n = 1483).** Pink = gram-positive bacteria, blue = gram-negative bacteria, green = fungi.

The distribution of blood culture isolates is summarised in Fig. 3 and Supplemental Table S3. Gram-negative bacteria predominated across sites (range: 53·8–91·0%), and overall (78·4%; 1163/1483). *Klebsiella* spp. was the most frequently isolated gram-negative bacteria (408/1483; 27·5%), followed by *Acinetobacter* spp. (261/1483; 17·6%), *Enterobacter* spp. (98/1483, 6·6%), and *Escherichia coli* (96/1483, 6·5%).

Gram-positive bacteria comprised 13·2% (196/1483) of isolates (site specific range: 4·0–36·8%). The most common gram-positives were *Staphylococcus aureus* (51/1483, 3·4%), Group B Streptococcus (33/1483, 2·2%), and *Enterococcus* spp. (30/1483, 2·0%), with variable contribution by site (Fig. 3).

Fungi accounted for 8·4% (124/1483, range: 0·0–12·9%) of isolates, with *Candida parapsilosis* (39/1483, 2·6%) and *Candida albicans* (21/1483, 1·4%) most frequently isolated. Of the 92/123 (66·7%) *Candida* isolates speciated, the majority 71/92 (77%) were non-albicans, with no *Candida auris* isolated.

### Antimicrobial susceptibility

Antimicrobial susceptibility data were available for 79·2% (1174/1483) of isolates, including 84·1% (978/1163) of gram-negatives, 56·6% (111/196) of gram-positives and 68·5% (85/124) of fungi (Supplemental Figure S1). Site-specific susceptibility data for each pathogen-antibiotic combination are detailed in Supplemental Tables S4–S23 and Supplemental Figures S2–S3.

**Fig. 4:**
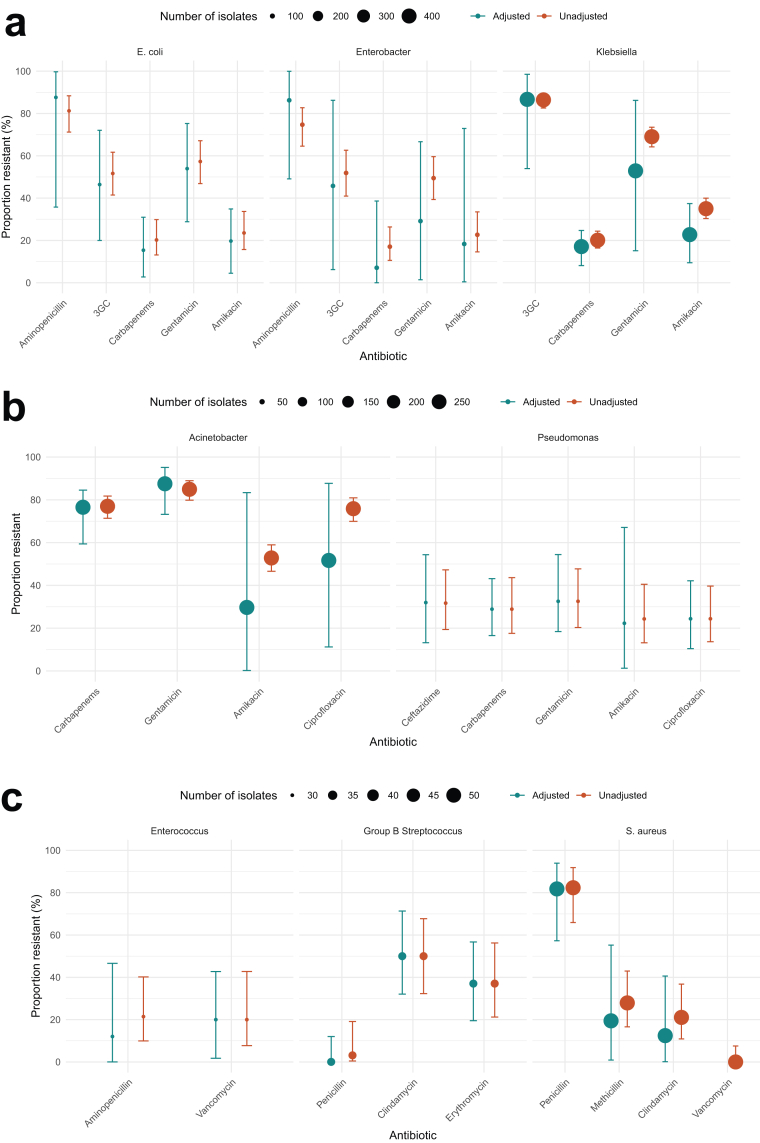
**Estimates of combined antimicrobial non-susceptibility across sites for key pathogen-antibiotic combinations before and after adjustment for clustering by site.** a) Enterobacterales, b) Gram-negative non-fermenters, c) Gram-positive bacteria. 3GC: Non-antipseudomonal 3rd Generation Cephalosporins.

Fig. 4 presents pooled estimates of key pathogen-antibiotic combinations across sites adjustment for clustering. Among the three most common Enterobacterales (Fig. 4a), non-susceptibility against 3GC and carbapenems for *Klebsiella* spp. was 86·7% (95% CI 54·0–98·5%) and 17·1% (95% CI 8·1–24·7%), for *E. coli* was 46·4% (95% CI 20·0–72·0%) and 15·4% (95% CI 2·7–31·0%), and for *Enterobacter* spp. was 45·8% (95% CI 6·2–86·2%) and 7·1% (95% CI 0·0–38·6%). Non-susceptibility against the aminoglycosides gentamicin and amikacin for *Klebsiella* spp. was 52·9% (95% CI 15·1–86·2%) and 22·7% (95% CI 9·4–37·4%), for *E. coli* was 53·9% (95% CI 28·8–75·3%) and 19·7% (95% CI 4·5–34·9%), and for *Enterobacter* spp. was 29·1% (95% CI 1·4–66·7%) and 18·3% (95% CI 0·4–72·9%). Meanwhile, non-susceptibility for *Acinetobacter* spp. against commonly used agents was considerable, with resistance to carbapenems of 76·5% (95% CI 59·4–84·5), gentamicin of 87·5% (95% CI 73·2–95·2%), and amikacin of 29·7% (95% CI 0·2–83·4%).

Among gram-positive bacteria (Fig. 4c), the prevalence of methicillin-resistant *S. aureus* was 19·4% (95% CI 0·9–55·2%), vancomycin resistant *Enterococcus* spp. was 20·0% (95% CI 1·7–42·7%), and erythromycin resistant Group B Streptococcus was 37·0% (10/27, 95% CI 19·5–56·7%).

Of the fungal isolates tested, no resistance was observed in the Philippines (n = 34), Sri Lanka (n = 2) or Vietnam (n = 9). Antifungal non-susceptibility was confined to Indonesia (Table 4) with pooled resistance for echinocandins of 11·1% (4/36), and for fluconazole of 9·4% (3/32). Resistant Candida spp. was all non-albicans species.

**Table 4:** Pooled antifungal resistance data for Indonesia by species and agent (n = 39).

Candida species	Antifungal agent					
	Fluconazole	Voriconazole	Amphotericin B	Caspofungin	Micafungin	Flucytosine
	n/N (%)					
*C. parapsilosis*	3/15 (20·0)	0/15 (0)	1/15 (6·7)	0/15 (0)	0/15 (0)	0/7 (0)
*C. albicans*	0/13 (0)	0/13 (0)	0/13 (0)	0/13 (0)	0/13 (0)	0/3 (0)
*C. glabrata*	0/3 (0)	0/5 (0)	0/5 (0)	4/5 (80·0)	3/5 (60·0)	0/2 (0)
*C. tropicalis*	0/1 (0)	0/1 (0)	0/1 (0)	0/1 (0)	0/1 (0)	0/1 (0)
*C. ciferii*	0/0 (0)	0/3 (0)	1/3 (33·3)	0/0 (0)	0/0 (0)	0/0 (0)
*C. guilliermondii*	0/0 (0)	0/2 (0)	0/2 (0)	0/2 (0)	1/2 (50·0)	0/2 (0)
**Total**	3/32 (9·4)	0/39 (0)	2/39 (5·1)	4/36 (11·1)	4/36 (11·1)	0/15 (0)

**Fig. 5:**
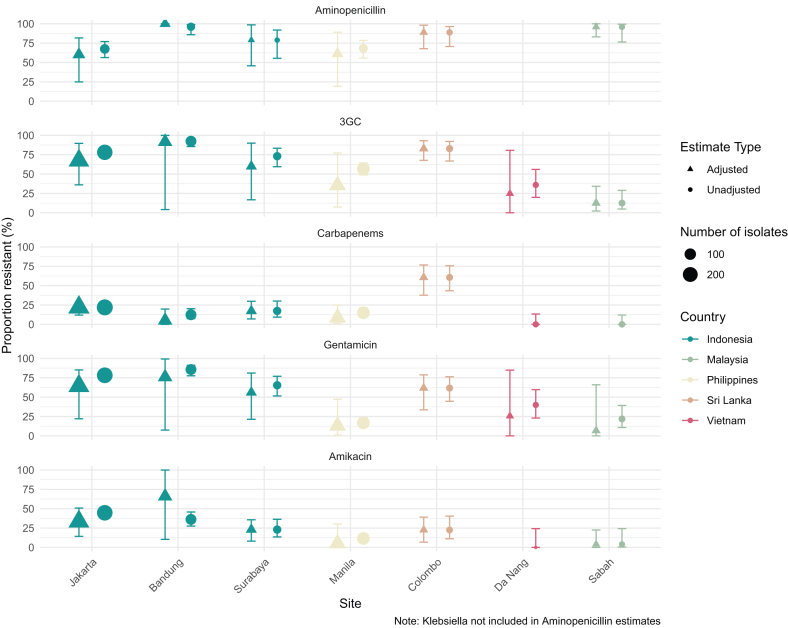
**Estimates of combined Enterobacterale antimicrobial non-susceptibility for key antimicrobials by site, before and after adjustment for clustering by species.** 3GC: Non-antipseudomonal 3rd Generation Cephalosporins.

Fig. 5 illustrates combined Enterobacterales non-susceptibility. Aminopenicillin non-susceptibility exceeded 50% across all sites (range: 60·3–100%). Resistance to gentamicin and alternative 3GC was greater than 50% in Indonesia (56·1–76·0% and 60·0–91·7%) and Colombo (61·8% and 82·9%) but remained lower in Manila, Da Nang, and Sabah. Amikacin resistance exhibited a similar trend, with the highest rates in Indonesia (23·1–66·0%) and Colombo (22·6%), while lower elsewhere. Carbapenem resistance was most prevalent in Colombo (60·6%) followed by Jakarta (21·9%) and Surabaya (17·3%), with less resistance in Manila, Bandung, Da Nang, and Sabah.

## Discussion

This study addressed important epidemiological gaps in understanding the AMR burden in neonatal sepsis in a high prevalence region of the world. By evaluating the causative agents of neonatal sepsis and non-susceptibility to commonly prescribed antibiotics in 10 clinical sites across five countries in South and Southeast Asia, we have revealed a marked predominance of gram-negative bacteria causing neonatal sepsis within the region, and alarming levels of non-susceptibility to commonly recommended empirical antibiotic regimens.

Our results align with recent observational studies conducted elsewhere in Asia and Africa, confirming that gram-negative bacteria are the major cause of neonatal sepsis in resource-constrained healthcare settings.^13^^,^^18^^,^^33^, ^34^, ^35^, ^36^ The predominance of *Klebsiella* spp., *Acinetobacter* spp., and *E. coli* as causative pathogens is consistent with other recent epidemiological data, but the relative contribution of *Enterobacter* spp. was higher than observed elsewhere. The high prevalence of *Klebsiella* spp. and *Acinetobacter* spp., which are frequently associated with nosocomial infections, underscores the potential role of very early horizontal transmission of bacteria causing neonatal sepsis in these settings.^13^^,^^18^^,^^28^ As observed across other LMIC settings, gram-positive bacteria were responsible for only a minority (13·2%) of culture-positive neonatal sepsis cases. *S. aureus* remained the most important gram-positive pathogen (after exclusion of coagulase-negative staphylococci) but accounted for a lower proportion of cases compared to previous multi-country studies in LMICs.

The leading bacterial sepsis pathogens demonstrated alarming levels of resistance to commonly recommended antimicrobial classes. Among gram-negatives, pooled susceptibility to the WHO-recommended antibiotics for neonatal sepsis: benzylpenicillin/ampicillin plus gentamicin or 3rd generation cephalosporins, were all less than fifty percent. These susceptibilities are as low as other observational studies in resource-constrained settings,^13^^,^^18^^,^^19^^,^^35^, ^36^, ^37^ supporting evidence that the current WHO-recommended empirical treatment regimens for neonatal sepsis do not provide sufficient coverage in urban hospitals in South and Southeast Asia.^23^ Updated empirical treatment guidelines adapted for settings with a high burden of multidrug-resistant infections are therefore urgently required to effectively treat infants, inform local stewardship programs, and harmonise global practices.

Susceptibility to amikacin and carbapenems, common alternative antimicrobials prescribed in high-burden AMR settings, was also concerningly low against both Enterobacterales and gram-negative non-fermenters. The high-burden of resistance to both first- and second-line agents highlights the urgent need for improved access to, and development of, novel antimicrobial agents which can effectively treat the growing number of infants in resource-constrained settings with multidrug-resistant infections.

The high burden of non-susceptibility in bacteria causing neonatal sepsis observed in this study occurred in large tertiary hospitals with well-established IPC and antimicrobial stewardship programs. Most sites have implemented multiple strategies to mitigate MRO transmission within their hospital settings but face high numbers of births and complex patients compounded by overcrowding, limited staffing and resource availability. The persistence of MRO infections despite these efforts, demonstrates the critical need for studies to evaluate effective interventions to reduce vertical and horizontal MRO transmission, alongside the regional (and global) spread of AMR.

An important finding of this study was the significant number of neonatal sepsis episodes caused by invasive fungal infections (8%; 124/1539). This highlights the need for enhanced mycology diagnostic capacity and improved fungal infection surveillance in LMIC settings. Unlike bacteria, the identification and susceptibility testing of *Candida* spp. remains an unavailable diagnostic tool in many laboratories worldwide; though pragmatic guidance has been established to enhance its implementation to enable improved surveillance of fungal infections in vulnerable populations, including neonates.^38^ The predominance of *C. parapsilosis* and *C. albicans* found here, supports their ranking on the WHO Fungal Priority Pathogen List,^39^ and recent inclusion in the Global Antimicrobial Resistance Surveillance System (GLASS).^36^ Pleasingly our data supports existing evidence that antifungal resistance in South and South-East Asia remains low.^40^^,^^41^ However, rise of antifungal resistance and cases of multidrug-resistant *C. auris* among neonates in other regions,^42^, ^43^, ^44^ underscores the need for robust fungal diagnostics and ongoing surveillance mechanisms in Southeast Asia.

Our findings should be interpreted within the context of some study limitations. Firstly, sites were predominantly large, tertiary-care urban hospitals with complex patients frequently requiring prolonged hospitalisation. These sites were sampled by a convenience rather than random selection method, focussing on a region of the world where published data pertaining to AMR in children is limited. The observed pathogen distribution and resistance profiles are therefore not nationally representative and may not be generalisable to other healthcare settings in the included countries, nor other countries within the region. However given the similarities in pathogen and antimicrobial resistance distributions across our sites, and concordance with other recent surveillance studies in resource-constrained settings, there is accumulating evidence of a high burden of gram-negative multidrug resistant blood stream infections in hospitalised neonates within the region.^13^^,^^18^^,^^35^ This highlights the need for further published surveillance data across the spectrum of healthcare settings in high-burden infant mortality countries, to enable more representative estimates of pathogen distribution and resistance patterns.

Secondly, blood culture data were collected retrospectively via microbiological databases which limited the evaluation of clinical correlates of reported infections. This precluded any analyses of associations between bacteraemia, potential risk factors and clinical outcomes. The establishment of prospective surveillance systems that incorporate clinical and microbiological data is therefore crucial to enable evaluation in trends over time, to inform empirical regimens and treatment decisions, assess the impact of interventions to reduce the burden of AMR and evaluate the impact of MRO infections on morbidity and mortality. Fortunately, these syndrome-specific surveillance programs have recently commenced across other clinical sites in Asia and Africa and are planned as a prospective activity at high burden sites identified within our study.^32^^,^^45^ Finally, laboratory capacity affected data availability from two sites; highlighting the urgent need to enhance microbiological capacity as part of a global strategy to address AMR. In the site in Sri Lanka, the identification of gram-negative Enterobacterales was limited to lactose fermentation-status for multiple isolates, while in one Vietnam site, species-level identification data were not available for most isolates. In other (private hospital) sites in Vietnam, collection of blood cultures prior to commencing empiric antibiotics was not routinely performed, yet this has now been implemented with recognition of the importance of this investigation in guiding appropriate antibiotic prescribing. These site limitations reduced the availability of antibiotic susceptibility data and may have biased pooled species-level estimates. These laboratory limitations are, unfortunately, commonplace in many LMICs and underscore the importance of strategies to support the accurate reporting of laboratory and research methods to enable robust published epidemiological estimates on neonatal sepsis and AMR.^43^^,^^44^

Despite these limitations, our study provides granular data in an under-represented global region to reveal clear evidence that neonatal sepsis in hospitalised infants in tertiary centres in South and Southeast Asia is predominantly caused by multidrug-resistant gram-negative bacteria. Our findings strengthen calls for updated, context-specific empirical treatment regimens against the contemporaneous causes of infection to reduce unnecessary neonatal morbidity and mortality. It also highlights the urgent need for clinical trials to assess alternative regimens that may provide better coverage,^25^ and the prioritised development of novel therapeutic agents to treat the growing number of infants with multi-drug resistant organisms.^12^^,^^29^^,^^46^^,^^47^ This data also underscores the pressing need to better understand the acquisition and transmission of MROs within hospital environments, so that targeted and efficacious interventions to reduce neonatal infections can be developed.

## Contributors

Conceptualisation: PCMW. Funding acquisition: PCMW. Methodology: PCMW and MS. Investigation: METV, NDP, RA, LK, GPSG, CHML, HTT, NXH, SMF, DS, TY, MU, NSC, NTKT, HNTT, TTCT, LTH, and NBH. Data curation: BFRD. Project administration: PCMW, BFRD, and MH. Writing – original draft: BFRD and PCMW. Writing – review & editing: all authors. All authors had access to the raw data. Data analysis was undertaken by BFRD, JB, TLS, and MJ. All authors reviewed the final manuscript, and PCMW had responsibility for the decision to submit for publication.

## Data sharing statement

Raw antimicrobial susceptibility data by site are available in Supplemental Tables S3–S22.

## Editor note

The Lancet Group takes a neutral position with respect to territorial claims in published maps and institutional affiliations.

## Declaration of interests

We declare no conflicts of interest.
