# Supplementary material for: Pathogen distribution and antimicrobial resistance among neonatal bloodstream infections in Southeast Asia: results from NeoSEAP, a multicentre retrospective study
Source: Lancet Reg Health West Pac. 2025 Sep 9;62:101617. doi: 10.1016/j.lanwpc.2025.101617 (PMC12455119; doi:10.1016/j.lanwpc.2025.101617)
Supplement: Supplemental Tables and Figures [file mmc1.docx]

**Appendix A. Supplementary Data**

**Table of Contents**

[Supplemental Table 1. Dates of site visits, resource availability, and infection prevention & control surveys 3](#_Toc191213868)

[Supplemental Table 2. List of pre-specified bacterial pathogens 3](#_Toc191213869)

[Supplemental Figure 1. Flow diagram outlining the total number of blood cultures processed, and the number of significant isolates (i.e excluding contaminants) included in the analyses across all sites. 5](#_Toc191213870)

[Supplemental Table 4. Non-susceptibility antibiogram of gram-negative bacteria for site 1 6](#_Toc191213871)

[Supplemental Table 5. Non-susceptibility antibiogram of gram-negative bacteria for site 2 7](#_Toc191213872)

[Supplemental Table 6. Non-susceptibility antibiogram of gram-negative bacteria for site 3. 8](#_Toc191213873)

[Supplemental Table 7. Non-susceptibility antibiogram of gram-negative bacteria for site 4. 9](#_Toc191213874)

[Supplemental Table 8. Non-susceptibility antibiogram of gram-negative bacteria for site 5. 10](#_Toc191213875)

[Supplemental Table 9. Non-susceptibility antibiogram of gram-negative bacteria for site 6 10](#_Toc191213876)

[Supplemental Table 10. Non-susceptibility antibiogram of gram-negative bacteria for site 7 11](#_Toc191213877)

[Supplemental Table 11. Non-susceptibility antibiogram of gram-positive bacteria for site 1 12](#_Toc191213878)

[Supplemental Table 12. Non-susceptibility antibiogram of gram-positive bacteria for site 2 12](#_Toc191213879)

[Supplemental Table 13. Non-susceptibility antibiogram of gram-positive bacteria for site 3 13](#_Toc191213880)

[Supplemental Table 14. Non-susceptibility antibiogram of gram-positive bacteria for site 4 13](#_Toc191213881)

[Supplemental Table 15. Non-susceptibility antibiogram of gram-positive bacteria for site 5 14](#_Toc191213882)

[Supplemental Table 16. Non-susceptibility antibiogram of gram-positive bacteria site 6 14](#_Toc191213883)

[Supplemental Table 17. Non-susceptibility antibiogram of gram-positive bacteria site 7 15](#_Toc191213884)

[Supplemental Table 18. Antifungal resistance profiles by species and agent for site 1 16](#_Toc191213885)

[Supplemental Table 19. Antifungal resistance profiles by species and agent for site 2 16](#_Toc191213886)

[Supplemental Table 20. Antifungal resistance profiles by species and agent for site 3 16](#_Toc191213887)

[Supplemental Table 21. Antifungal resistance profiles by species and agent for site 4 17](#_Toc191213888)

[Supplemental Table 22. Antifungal resistance profiles by species and agent for site 5 17](#_Toc191213889)

[Supplemental Table 23. Antifungal resistance profiles by species and agent for site 7 17](#_Toc191213890)

[Supplemental Figure 2. Unadjusted antimicrobial non-susceptibility for Enterobacterales by site, pathogen and antimicrobial 18](#_Toc191213891)

[Supplemental Figure 3. Unadjusted antimicrobial non-susceptibility for Gram-negative non-fermenters by site, pathogen and antimicrobial 19](#_Toc191213892)

[Supplemental Figure 4. Unadjusted antimicrobial non-susceptibility for gram-positive bacteria by site, pathogen and antimicrobial 20](#_Toc191213893)

| Supplemental Table 1. Dates of site visits, resource availability, and infection prevention & control surveys | | | |
| --- | --- | --- | --- |
| **Site** | **Country** | **Survey Date** | **Site visit date for data confirmation** |
| 1 | Indonesia | October 2022 | June 2022, August 2023 |
| 2 | Indonesia | January 2022 | June 2022 |
| 3 | Indonesia | September 2022 | June 2022 |
| 4 | The Philippines | October 2022 | March 2022, May 2023 |
| 5 | Sri Lanka | December 2021 | March 2022 |
| 6 | Malaysia | April 2023 | No visit |
| 7 | Vietnam | July 2022 | February 2023 |
| 8 | Vietnam | January 2023 | February 2023 |
| 9 | Vietnam | February 2023 | February 2023 |
| 10 | Vietnam | January 2023 | No visit |

| Supplemental Table 2. List of pre-specified bacterial pathogens | |
| --- | --- |
| **Gram-positive bacteria** | **Gram-negative bacteria** |
| *Staphylococcus aureus*  Coagulase-negative *Staphylococcus* spp.  *Enterococcus* spp.  *Streptococcus agalactiae* (Group B Streptococcus)  *Streptococcus pyogenes* (Group A Streptococcus)  *Streptococcus pneumoniae* | **Enterobacterales**  *E. coli*  *Klebsiella* spp.  *Enterobacter* spp.  *Citrobacter* spp.  *Serratia* spp.  *Proteus* spp.  *Raoutella* spp.  *Haemophilus influenzae*  *Salmonella enterica* var. typhi  *Non-typhoid* Salmonella spp. |
|  | **Non-fermenting gram-negative bacilli**  *Acinetobacter* spp.  *Pseudomonas* spp. |

| **Supplemental Table 3. Frequency of isolates in neonatal blood cultures, by site (n=1,483)** | | | | |  |  |  |  |  |
| --- | --- | --- | --- | --- | --- | --- | --- | --- | --- |
| **Country** |  |  | **Indonesia** |  | **Philippines** | **Sri Lanka** | **Malaysia** | **Vietnam** | **Total** |
| **Site** | **Pathogen** | **1** | **2** | **3** | **4** | **5** | **6** | **7** |  |
| **Gram negative**  **(n) (%)** | *Acinetobacter* spp· | 90 | 61 | 11 | 67 | 30 | 1 | 1 | 261 (17.6) |
|  | *Citrobacter* spp· | 2 | 0 | 0 | 0 | 0 | 0 | 0 | 2 (0.1) |
|  | *E· coli* | 19 | 31 | 7 | 14 | 1 | 15 | 9 | 96 (6.5) |
|  | *Enterobacter* spp· | 62 | 5 | 6 | 9 | 7 | 8 | 1 | 98 (6.6) |
|  | *Klebsiella* spp· | 204 | 64 | 33 | 92 | 2 | 7 | 6 | 408 (27.5) |
|  | Lactose-fermenting (LF) coliforms | 0 | 0 | 0 | 0 | 14 | 0 | 0 | 14 (0.9) |
|  | Non-lactose-fermenting (NLF) coliforms | 0 | 0 | 0 | 0 | 11 | 0 | 0 | 11 (0.7) |
|  | Non-typhoid Salmonella spp· | 0 | 21 | 0 | 0 | 0 | 0 | 0 | 21 (1.4) |
|  | *Proteus* spp· | 1 | 2 | 0 | 0 | 0 | 1 | 0 | 4 (0.3) |
|  | *Pseudomonas* spp· | 17 | 6 | 4 | 8 | 5 | 5 | 1 | 46 (3.1) |
|  | *S· typhi* | 0 | 2 | 0 | 1 | 0 | 0 | 0 | 3 (0.2) |
|  | *Serratia* spp· | 3 | 9 | 6 | 40 | 1 | 1 | 9 | 69 (4.7) |
|  | Other | 8 | 17 | 1 | 0 | 5 | 5 | 94 | 130 (8.8) |
|  | **Total** | 406 (91·0) | 218 (82·0) | 68 (74·7) | 231 (82·8) | 76 (70·4) | 43 (63·2) | 121 (53·8) | 1,163 (78.4) |
| **Gram positive**  **(n) (%)** | *Enterococcus* spp· | 9 | 7 | 0 | 7 | 6 | 1 | 0 | 30 (2.0) |
|  | Group A Streptococcus | 1 | 1 | 0 | 0 | 1 | 0 | 0 | 3 (0.2) |
|  | Group B Streptococcus | 1 | 11 | 0 | 2 | 12 | 5 | 2 | 33 (2.2) |
|  | *S· aureus* | 7 | 7 | 2 | 5 | 7 | 16 | 7 | 51 (3.4) |
|  | *S· pneumoniae* | 0 | 0 | 0 | 0 | 0 | 1 | 0 | 1 (0.1) |
|  | Other | 0 | 0 | 10 | 0 | 0 | 2 | 66 | 78 (5.3) |
|  | **Total** | 18 (4·0) | 26 (9·8) | 12 (13·2) | 14 (5·0) | 26 (24·1) | 25 (36·8) | 75 (33·3) | 196 (13.2) |
| **Fungal**  **(n) (%)** | *C· albicans* | 3 | 11 | 2 | 3 | 1 | 0 | 1 | 21 (1.4) |
|  | *C· glabrata* | 0 | 3 | 2 | 1 | 0 | 0 | 0 | 6 (0.4) |
|  | *C· parapsilosis* | 5 | 8 | 2 | 16 | 0 | 0 | 8 | 39 (2.6) |
|  | *C· tropicalis* | 0 | 0 | 1 | 10 | 0 | 0 | 0 | 11 (0.7) |
|  | *C· ciferii* | 3 | 0 | 0 | 0 | 0 | 0 | 0 | 3 (0.2) |
|  | *C· famata* | 6 | 0 | 3 | 0 | 0 | 0 | 0 | 9 (0.6) |
|  | *C· guilliermondii* | 2 | 0 | 0 | 0 | 0 | 0 | 0 | 2 (0.1) |
|  | *C· pelliculosa* | 1 | 0 | 0 | 0 | 0 | 0 | 0 | 1 (0.1) |
|  | *Candida spp·* (other or not speciated) | 1 | 0 | 1 | 4 | 5 | 0 | 20 | 31 (2.1) |
|  | Other fungi (*Kodamaea ohmeri*) | 1 | 0 | 0 | 0 | 0 | 0 | 0 | 1 (0.1) |
|  | **Total** | 22 (4·9) | 22 (8·3) | 11 (12·1) | 34 (12·2) | 6 (5·6) | 0 (0) | 29 (12·9) | 124 (8.4) |

# Supplemental Figure 1. Flow diagram of the total number of blood cultures processed, and the number of significant (non-contaminant) isolates included in the analyses across all sites.

NB: Positive cultures from the same patient with the same organism collected within four weeks were considered to occur as part of the same infectious episode, and were removed as duplicates.

| Supplemental Table 4. Non-susceptibility antibiogram of gram-negative bacteria for site 1 | | | | | | | | | | | | | | | | | | | | | | | | |
| --- | --- | --- | --- | --- | --- | --- | --- | --- | --- | --- | --- | --- | --- | --- | --- | --- | --- | --- | --- | --- | --- | --- | --- | --- |
|  | ***Acinetobacter* spp.** | | | ***Citrobacter* spp.** | | | ***E. coli*** | | | ***Enterobacter* spp.** | | | ***Klebsiella* spp.** | | | ***Proteus* spp.** | | | ***Pseudomonas* spp.** | | | ***Serratia* spp.** | | |
|  | **N** | **n** | **%** | **N** | **n** | **%** | **N** | **n** | **%** | **N** | **n** | **%** | **N** | **n** | **%** | **N** | **n** | **%** | **N** | **n** | **%** | **N** | **n** | **%** |
| 3GC~ |  | NR* |  | 2 | 0 | 0 | 19 | 9 | 47.4 | 44 | 26 | 59.1 | 183 | 158 | 86.3 | 1 | 1 | 100 |  | NR |  | 2 | 2 | 100 |
| Amikacin | 82 | 63 | 76.8 | 2 | 0 | 0 | 17 | 6 | 35.3 | 47 | 10 | 21.3 | 187 | 98 | 52.4 | 1 | 0 | 0 | 15 | 3 | 20 | 3 | 1 | 33.3 |
| Aminopenicillin |  | NR |  | 2 | 0 | 0 | 17 | 8 | 47.1 | 55 | 42 | 76.4 |  |  |  | 1 | 1 | 100 |  | NR |  | 2 | 1 | 50 |
| Amoxicillin-Clavulanate |  | NR |  | 2 | 0 | 0 | 18 | 6 | 33.3 | 56 | 51 | 91.1 | 194 | 119 | 61.3 | 1 | 1 | 100 |  | NR |  | 3 | 3 | 100 |
| Carbapenems | 85 | 68 | 80.0 | 2 | 0 | 0 | 17 | 5 | 29.4 | 56 | 8 | 14.3 | 195 | 47 | 24.1 | 1 | 0 | 0 | 17 | 5 | 29.4 | 3 | 0 | 0 |
| Cefalexin |  | NR |  | 0 | 0 |  | 0 | 0 |  | 3 | 0 | 0 | 0 | 0 |  | 0 | 0 |  |  | NR |  | 0 | 0 |  |
| Cefepime | 50 | 42 | 84.0 | 1 | 0 | 0 | 10 | 3 | 30 | 33 | 12 | 36.4 | 118 | 98 | 83.1 | 1 | 0 | 0 | 8 | 0 | 0 | 2 | 0 | 0 |
| Ceftazidime | 66 | 55 | 83.3 | 2 | 0 | 0 | 13 | 8 | 61.5 | 38 | 17 | 44.7 | 156 | 132 | 84.6 | 1 | 1 | 100 | 13 | 2 | 15.4 | 2 | 2 | 100 |
| Ciprofloxacin | 73 | 59 | 80.8 | 2 | 0 | 0 | 17 | 4 | 23.5 | 39 | 11 | 28.2 | 175 | 118 | 67.4 | 1 | 1 | 100 | 13 | 2 | 15.4 | 2 | 0 | 0 |
| Co-trimoxazole | 29 | 9 | 31.0 | 0 | 0 |  | 4 | 1 | 25.0 | 26 | 6 | 23.1 | 94 | 64 | 68.1 | 0 | 0 |  |  | NR |  | 1 | 1 | 100 |
| Gentamicin | 77 | 66 | 85.7 | 2 | 0 | 0 | 17 | 9 | 52.9 | 55 | 33 | 60.0 | 185 | 161 | 87.0 | 1 | 0 | 0 | 15 | 3 | 20 | 2 | 2 | 100 |
| Piperacillin-Tazobactam | 84 | 68 | 81.0 | 2 | 0 | 0 | 0 | 0 |  | 55 | 16 | 29.1 | 0 | 0 |  | 1 | 0 | 0 | 16 | 3 | 18.8 | 3 | 0 | 0 |
| Tigecycline | 82 | 9 | 11.0 | 1 | 0 | 0 | 16 | 0 | 0 | 54 | 0 | 0 | 190 | 5 | 2.63 | 1 | 0 | 0 | 13 | 10 | 76.9 | 1 | 0 | 0 |
| *Not reported because of intrinsic resistance or limited clinical use. Antimicrobials not used for susceptibility tested were omitted from the table. ~3^rd^ generation cephalosporin. N: Number tested; n: Number non-susceptible. | | | | | | | | | | | | | | | | | | | | | | | | |

| Supplemental Table 5. Non-susceptibility antibiogram of gram-negative bacteria for site 2 | | | | | | | | | | | | | | | | | | | | | | | | | | | |
| --- | --- | --- | --- | --- | --- | --- | --- | --- | --- | --- | --- | --- | --- | --- | --- | --- | --- | --- | --- | --- | --- | --- | --- | --- | --- | --- | --- |
|  | ***Acinetobacter* spp.** | | | ***E. coli*** | | | ***Enterobacter* spp.** | | | ***Klebsiella* spp.** | | | **Non-typhoid *Salmonella* spp.** | | | ***Proteus* spp.** | | | ***Pseudomonas* spp.** | | | ***S. typhi*** | | | ***Serratia* spp.** | | |
|  | **N** | **n** | **(%)** | **N** | **n** | **(%)** | **N** | **n** | **(%)** | **N** | **n** | **(%)** | **N** | **n** | **(%)** | **N** | **n** | **(%)** | **N** | **n** | **(%)** | **N** | **n** | **(%)** | **N** | **n** | **(%)** |
| 3GC~ |  | NR* |  | 26 | 23 | 88.5 | 5 | 5 | 100 | 53 | 53 | 100 | 13 | 13 | 100 | 1 | 0 | 0 |  | NR |  | 1 | 0 | 0 | 6 | 3 | 50 |
| Amikacin | 57 | 34 | 59.7 | 26 | 9 | 34.6 | 5 | 5 | 100 | 53 | 9 | 17.0 | 13 | 13 | 100 | 1 | 0 | 0 | 5 | 1 | 20.0 | 1 | 1 | 100 | 6 | 1 | 16.7 |
| Aminopenicillin NR | | | | 26 | 26 | 100 | 5 | 5 | 100 |  | NR |  | 13 | 13 | 100 | 1 | 0 | 0 |  | NR |  | 1 | 0 | 0 | 6 | 6 | 100 |
| Amoxicillin-Clavulanate NR | | | | 26 | 17 | 65.4 | 5 | 5 | 100 | 53 | 53 | 100 |  | NR |  | 1 | 0 | 0 |  | NR |  |  | NR |  | 6 | 6 | 100 |
| Carbapenems | 57 | 47 | 82.5 | 26 | 9 | 34.6 | 5 | 0 | 0 | 53 | 4 | 7.5 | 13 | 0 | 0 | 1 | 0 | 0 | 5 | 2 | 40.0 | 1 | 0 | 0 | 6 | 0 | 0 |
| Cefepime | 57 | 55 | 96.5 | 26 | 12 | 46.2 | 5 | 5 | 100 | 53 | 24 | 45.3 |  | NR |  | 1 | 0 | 0 | 5 | 5 | 100 |  | NR |  | 6 | 0 | 0 |
| Ceftazidime | 57 | 55 | 96.5 | 26 | 19 | 73.1 | 5 | 5 | 100 | 53 | 53 | 100 |  | NR |  | 1 | 0 | 0 | 5 | 3 | 60.0 |  | NR |  | 6 | 3 | 50.0 |
| Ciprofloxacin | 57 | 52 | 91.2 | 26 | 9 | 34.6 | 5 | 5 | 100 | 53 | 24 | 45.3 | 13 | 13 | 100 | 1 | 0 | 0 | 5 | 2 | 40.0 | 1 | 1 | 100 | 6 | 3 | 50.0 |
| Co-trimoxazole | 57 | 18 | 31.6 | 26 | 11 | 42.3 | 5 | 4 | 80 | 53 | 27 | 50.9 | 13 | 6 | 46.2 | 1 | 0 | 0 |  | NR |  | 1 | 0 | 0 | 6 | 1 | 16.7 |
| Gentamicin | 57 | 55 | 96.5 | 26 | 23 | 88.5 | 5 | 0 | 0 | 53 | 50 | 94.3 | 13 | 13 | 100 | 1 | 0 | 0 | 5 | 3 | 60.0 | 1 | 1 | 100 | 6 | 3 | 50 |
| Piperacillin-Tazobactam | 57 | 55 | 96.5 |  | NR |  | 5 | 2 | 40.0 |  |  |  |  | NR |  | 1 | 0 | 0 | 5 | 3 | 60.0 |  | NR |  | 6 | 1 | 16.7 |
| Tigecycline | 57 | 8 | 14.0 | 26 | 0 | 0 | 5 | 2 | 40 | 53 | 3 | 5.7 |  | NR |  | 1 | 0 | 0 | 5 | 3 | 60.0 |  | NR |  | 6 | 0 | 0 |
| Trimethoprim NR | | | | 26 | 11 | 42.3 | 5 | 4 | 80 | 53 | 27 | 50.9 |  | NR |  | 1 | 0 | 0 |  | NR |  |  | NR |  | 6 | 1 | 16.7 |

*Not reported because of intrinsic resistance or limited clinical use. Antimicrobials not used for susceptibility tested were omitted from the table. ~3^rd^ generation cephalosporin. N: Number tested; n: Number non-susceptible.

| Supplemental Table 6. Non-susceptibility antibiogram of gram-negative bacteria for site 3. | | | | | | | | | | | | | | | | | | |
| --- | --- | --- | --- | --- | --- | --- | --- | --- | --- | --- | --- | --- | --- | --- | --- | --- | --- | --- |
|  | ***Acinetobacter* spp.** | | | ***E. coli*** | | | ***Enterobacter* spp.** | | | ***Klebsiella* spp.** | | | ***Pseudomonas* spp.** | | | ***Serratia* spp.** | | |
|  | **N** | **n** | **%** | **N** | **n** | **%** | **N** | **n** | **%** | **N** | **n** | **%** | **N** | **n** | **%** | **N** | **n** | **%** |
| 3GC~ |  | NR* |  | 7 | 4 | 57.1 | 6 | 3 | 50.0 | 33 | 30 | 90.9 |  | NR |  | 6 | 1 | 16.7 |
| Amikacin | 11 | 8 | 72.7 | 7 | 2 | 28.6 | 6 | 1 | 16.7 | 33 | 9 | 27.3 | 4 | 0 | 0 | 6 | 0 | 0 |
| Aminopenicillin NR | | |  | 7 | 7 | 100 | 6 | 4 | 66.7 |  | NR |  |  | NR |  | 6 | 4 | 66.7 |
| Amoxicillin-Clavulanate NR | | | | 7 | 4 | 57.1 | 6 | 4 | 66.7 | 33 | 26 | 78.8 |  | NR |  | 6 | 4 | 66.7 |
| Carbapenems | 11 | 6 | 54.5 | 7 | 2 | 28.6 | 6 | 1 | 16.7 | 33 | 6 | 18.2 | 4 | 1 | 25.0 | 6 | 0 | 0 |
| Cefalexin | | NR |  | 7 | 0 | 0 | 6 | 0 | 0 |  | NR |  |  | NR |  | 6 | 0 | 0 |
| Cefepime | 11 | 10 | 90.9 | 7 | 6 | 85.7 | 6 | 6 | 100 | 33 | 30 | 90.9 | 4 | 2 | 50.0 | 6 | 2 | 33.3 |
| Ceftazidime | 11 | 10 | 90.9 | 7 | 4 | 57.1 | 6 | 5 | 83.3 | 33 | 30 | 90.9 | 4 | 2 | 50.0 | 6 | 1 | 16.7 |
| Ciprofloxacin | 11 | 0 | 0 | 7 | 0 | 0 | 6 | 0 | 0 | 33 | 0 | 0 | 4 | 0 | 0 | 6 | 0 | 0 |
| Co-trimoxazole | 11 | 0 | 0 | 7 | 0 | 0 | 6 | 0 | 0 | 33 | 0 | 0 |  | NR |  | 6 | 0 | 0 |
| Fosfomycin | | NR |  | 7 | 1 | 14.3 | 6 | 1 | 16.7 | 33 | 6 | 18.2 | 4 | 1 | 25.0 | 6 | 0 | 0 |
| Gentamicin | 11 | 10 | 90.9 | 7 | 4 | 57.1 | 6 | 3 | 50 | 33 | 26 | 78.8 | 4 | 2 | 50.0 | 6 | 1 | 16.7 |
| Minocycline | 11 | 0 | 0 |  | NR |  |  | NR |  |  | NR |  |  | NR |  |  | NR |  |
| Netilmicin | 11 | 1 | 9.1 | 7 | 0 | 0 | 6 | 1 | 16.7 | 33 | 1 | 3.0 | 4 | 1 | 25.0 | 6 | 0 | 0 |
| Norfloxacin | | NR |  |  | NR |  |  | NR |  | 33 | 0 | 0 |  | NR |  |  | NR |  |
| Piperacillin-Tazobactam | 11 | 9 | 81.8 |  | NR |  | 6 | 2 | 33.3 |  | NR |  | 4 | 0 | 0 | 6 | 1 | 16.7 |
| Tigecycline | 11 | 1 | 9.1 | 7 | 1 | 14.3 | 6 | 0 | 0 | 33 | 19 | 57.6 | 4 | 2 | 50.0 | 6 | 0 | 0 |
| Tobramycin | 11 | 0 | 0 | 7 | 0 | 0 | 6 | 0 | 0 | 33 | 0 | 0 | 4 | 0 | 0 | 6 | 0 | 0 |
| Trimethoprim NR | | |  | 7 | 0 | 0 | 6 | 0 | 0 | 33 | 0 | 0 |  | NR |  | 6 | 0 | 0 |

*Not reported because of intrinsic resistance or limited clinical use. Antimicrobials not used for susceptibility tested were omitted from the table. ~3^rd^ generation cephalosporin. N: Number tested; n: Number non-susceptible.

| Supplemental Table 7. Non-susceptibility antibiogram of gram-negative bacteria for site 4. | | | | | | | | | | | | | | | | | | |
| --- | --- | --- | --- | --- | --- | --- | --- | --- | --- | --- | --- | --- | --- | --- | --- | --- | --- | --- |
|  | ***Acinetobacter* spp.** | | | ***E. coli*** | | | ***Enterobacter* spp.** | | | ***Klebsiella* spp.** | | | ***Pseudomonas* spp.** | | | ***Serratia* spp.** | | |
|  | **N** | **n** | **(%)** | **N** | **n** | **(%)** | **N** | **n** | **(%)** | **N** | **n** | **(%)** | **N** | **n** | **(%)** | **N** | **n** | **(%)** |
| 3GC~ |  | NR* |  | 14 | 4 | 28.6 | 9 | 2 | 22.2 | 92 | 77 | 83.7 |  | NR |  | 40 | 5 | 12.5 |
| Amikacin | 67 | 0 | 0 | 14 | 3 | 21.4 | 9 | 0 | 0 | 92 | 15 | 16.3 | 3 | 3 | 100 | 40 | 0 | 0 |
| Aminopenicillin |  | NR |  | 14 | 10 | 71.4 | 9 | 2 | 22.2 |  | NR |  |  | NR |  | 40 | 31 | 77.5 |
| Amoxicillin-Clavulanate |  | NR |  | 14 | 6 | 42.9 | 9 | 0 | 0 | 92 | 77 | 83.7 |  | NR |  | 40 | 31 | 77.5 |
| Carbapenems | 67 | 46 | 68.7 | 14 | 2 | 14.3 | 9 | 0 | 0 | 92 | 20 | 21.7 | 8 | 3 | 37.5 | 40 | 1 | 2.5 |
| Cefepime | 67 | 50 | 74.6 | 14 | 3 | 21.4 | 9 | 2 | 22.2 | 92 | 77 | 83.7 | 8 | 4 | 50 | 40 | 2 | 5 |
| Ceftazidime | 67 | 50 | 74.6 | 14 | 3 | 21.4 | 9 | 2 | 22.2 | 92 | 77 | 83.7 | 8 | 3 | 37.5 | 40 | 1 | 2.5 |
| Ciprofloxacin | 67 | 45 | 67.2 | 14 | 4 | 28.6 | 9 | 1 | 11.1 | 92 | 35 | 38.0 | 8 | 3 | 37.5 | 40 | 0 | 0 |
| Co-trimoxazole | 67 | 16 | 23.9 | 14 | 5 | 35.7 | 9 | 2 | 22.2 | 92 | 43 | 46.7 |  | NR |  | 40 | 1 | 2.5 |
| Gentamicin | 67 | 48 | 71.6 | 14 | 3 | 21.4 | 9 | 3 | 33.3 | 92 | 20 | 21.7 | 8 | 3 | 37.5 | 40 | 0 | 0 |
| Netilmicin | 0 | 0 | 0 | 0 | 0 |  | 0 | 0 |  | 0 | 0 |  | 8 | 0 | 0 | 40 | 0 | 0 |
| Piperacillin-Tazobactam | 67 | 50 | 74.6 |  | NR |  | 9 | 0 | 0 |  | NR |  | 8 | 3 | 37.5 | 40 | 0 | 0 |
| Tobramycin | 67 | 11 | 16.4 | 0 | 0 |  | 9 | 0 | 0 | 92 | 2 | 2.2 | 8 | 0 | 0 | 40 | 0 | 0 |

*Not reported because of intrinsic resistance or limited clinical use. Antimicrobials not used for susceptibility tested were omitted from the table. ~3^rd^ generation cephalosporin. N: Number tested; n: Number non-susceptible.

| Supplemental Table 8. Non-susceptibility antibiogram of gram-negative bacteria for site 5. | | | | | | | | | | | | | | | | | | | | | | | | |
| --- | --- | --- | --- | --- | --- | --- | --- | --- | --- | --- | --- | --- | --- | --- | --- | --- | --- | --- | --- | --- | --- | --- | --- | --- |
|  | ***Acinetobacter* spp.** | | | ***E. coli*** | | | ***Enterobacter* spp.** | | | ***Klebsiella* spp.** | | | **Lactose-fermenting (LF) coliforms** | | | **Non-lactose-fermenting (NLF) coliforms** | | | ***Pseudomonas* spp.** | | | ***Serratia* spp.** | | |
|  | **N** | **n** | **(%)** | **N** | **n** | **(%)** | **N** | **n** | **(%)** | **N** | **n** | **(%)** | **N** | **n** | **(%)** | **N** | **n** | **(%)** | **N** | **n** | **(%)** | **N** | **n** | **(%)** |
| 3GC~ |  | NR* |  | 1 | 0 | 0 | 6 | 5 | 83.3 | 2 | 2 | 100 | 14 | 12 | 85.7 | 11 | 9 | 81.8 |  | NR |  | 1 | 1 | 100 |
| Amikacin | 29 | 26 | 89.7 | 1 | 0 | 0 | 7 | 1 | 14.3 | 2 | 0 | 0 | 11 | 4 | 36.4 | 9 | 2 | 22.2 | 5 | 2 | 40.0 | 1 | 0 | 0 |
| Aminopenicillin NR | | | | 1 | 0 | 0 | 4 | 4 | 100 |  | NR |  | 11 | 10 | 90.9 | 10 | 9 | 90 |  | NR |  | 1 | 1 | 100 |
| Amoxicillin-Clavulanate NR | | | | 1 | 0 | 0 | 6 | 6 | 100 | 2 | 2 | 100 | 13 | 12 | 92.3 | 10 | 9 | 90.0 |  | NR |  | 1 | 1 | 100 |
| Carbapenems | 30 | 27 | 90.0 | 1 | 0 | 0 | 7 | 6 | 85.7 | 2 | 1 | 50 | 11 | 6 | 54.6 | 11 | 7 | 63.6 | 5 | 1 | 20.0 | 1 | 0 | 0 |
| Ceftazidime | 27 | 27 | 100 | 0 | 0 |  | 6 | 5 | 83.3 | 2 | 2 | 100 | 9 | 7 | 77.8 | 7 | 5 | 71.4 | 5 | 3 | 60.0 | 1 | 1 | 100 |
| Ciprofloxacin | 22 | 20 | 90.9 | 1 | 0 | 0 | 7 | 6 | 85.7 | 1 | 1 | 100 | 11 | 5 | 45.4 | 10 | 6 | 60 | 5 | 2 | 40.0 | 1 | 1 | 100 |
| Co-trimoxazole | 26 | 24 | 92.3 | 0 | 0 |  | 7 | 4 | 57.1 | 2 | 0 | 0 | 8 | 4 | 50 | 6 | 5 | 83.3 |  | NR |  | 1 | 0 | 0 |
| Gentamicin | 25 | 23 | 92.0 | 1 | 0 | 0 | 7 | 6 | 85.7 | 2 | 0 | 0 | 14 | 9 | 64.3 | 9 | 5 | 55.6 | 5 | 2 | 40.0 | 1 | 1 | 100 |
| Netilmicin | 17 | 15 | 88.2 | 1 | 0 | 0 | 1 | 0 | 0 | 0 | 0 |  | 7 | 5 | 71.4 | 6 | 3 | 50.0 | 5 | 3 | 60.0 | 0 | 0 |  |
| Piperacillin-Tazobactam | 0 | 0 |  |  | NR |  | 0 | 0 |  |  | NR |  | 0 | 0 |  | 0 | 0 |  | 0 | 0 |  | 1 | 0 | 0 |

*Not reported because of intrinsic resistance or limited clinical use. Antimicrobials not used for susceptibility tested were omitted from the table. ~3^rd^ generation cephalosporin.

| Supplemental Table 9. Non-susceptibility antibiogram of gram-negative bacteria for site 6 | | | | | | | | | | | | | | | | | | | | | |
| --- | --- | --- | --- | --- | --- | --- | --- | --- | --- | --- | --- | --- | --- | --- | --- | --- | --- | --- | --- | --- | --- |
|  | ***Acinetobacter* spp.** | | | ***E. coli*** | | | ***Enterobacter* spp.** | | | ***Klebsiella* spp.** | | | ***Proteus* spp.** | | | ***Pseudomonas* spp.** | | | ***Serratia* spp.** | | |
|  | **N** | **n** | **(%)** | **N** | **n** | **(%)** | **N** | **n** | **(%)** | **N** | **n** | **(%)** | **N** | **n** | **(%)** | **N** | **n** | **(%)** | **N** | **n** | **(%)** |
| 3GC~ |  | NR* |  | 15 | 2 | 13.3 | 8 | 0 | 0 | 7 | 1 | 14.3 | 1 | 0 | 0 |  | NR |  | 1 | 1 | 100 |
| Amikacin | 1 | 0 | 0 | 15 | 0 | 0 | 0 | 0 |  | 7 | 1 | 14.3 | 1 | 0 | 0 | 5 | 0 | 0 | 1 | 0 | 0 |
| Aminopenicillin NR | | |  | 15 | 14 | 93.3 | 8 | 8 | 100 |  | NR |  | 1 | 1 | 100 |  | NR |  | 1 | 1 | 100 |
| Amoxicillin-clavulanate NR | | | | 15 | 7 | 46.7 | 8 | 6 | 75.0 | 7 | 5 | 71.4 | 1 | 1 | 100 |  | NR |  | 1 | 1 | 100 |
| Carbapenems | 1 | 0 | 0 | 15 | 0 | 0 | 4 | 0 | 0 | 7 | 0 | 0 | 1 | 0 | 0 | 5 | 0 | 0 | 1 | 0 | 0 |
| Cefepime | 0 | 0 |  | 15 | 1 | 6.7 | 8 | 0 | 0 | 7 | 1 | 14.3 | 1 | 0 | 0 | 5 | 0 | 0 | 1 | 1 | 100 |
| Ceftazidime | 1 | 0 | 0 | 15 | 1 | 6.7 | 8 | 0 | 0 | 7 | 1 | 14.3 | 1 | 0 | 0 | 5 | 0 | 0 | 1 | 1 | 100 |
| Ciprofloxacin | 1 | 0 | 0 | 15 | 7 | 46.7 | 8 | 1 | 12.5 | 7 | 0 | 0 | 1 | 0 | 0 | 5 | 0 | 0 | 1 | 0 | 0 |
| Co-trimoxazole | 0 | 0 |  | 15 | 10 | 66.7 | 8 | 0 | 0 | 7 | 2 | 28.6 | 1 | 0 | 0 |  | NR |  | 1 | 0 | 0 |
| Gentamicin | 1 | 1 | 100 | 15 | 6 | 40.0 | 8 | 0 | 0 | 7 | 0 | 0 | 1 | 0 | 0 | 5 | 0 | 0 | 1 | 1 | 100 |
| Piperacillin-tazobactam | 1 | 0 | 0 |  | NR |  | 8 | 0 | 0 |  | NR |  | 1 | 0 | 0 | 5 | 0 | 0 | 1 | 1 | 100 |

*Not reported because of intrinsic resistance or limited clinical use. Antimicrobials not used for susceptibility tested were omitted from the table. ~3^rd^ generation cephalosporin. N: Number tested; n: Number non-susceptible.

| Supplemental Table 10. Non-susceptibility antibiogram of gram-negative bacteria for site 7 | | | | | | | | | | | | | | | | | | |
| --- | --- | --- | --- | --- | --- | --- | --- | --- | --- | --- | --- | --- | --- | --- | --- | --- | --- | --- |
|  | ***Acinetobacter* spp.** | | | **E. coli** | | | ***Enterobacter* spp.** | | | ***Klebsiella* spp.** | | | ***Pseudomonas* spp.** | | | ***Serratia* spp.** | | |
|  | **N** | **n** | **(%)** | **N** | **n** | **(%)** | **N** | **n** | **(%)** | **N** | **n** | **(%)** | **N** | **n** | **(%)** | **N** | **n** | **(%)** |
| 3GC~ |  | NR* |  | 9 | 5 | 55.6 | 1 | 0 | 0 | 6 | 4 | 66.67 |  | NR |  | 9 | 0 | 0 |
| Amikacin | 1 | 0 | 0 | 5 | 0 | 0 | 1 | 0 | 0 | 3 | 0 | 0 | 0 | 0 |  | 3 | 0 | 0 |
| Amoxicillin-clavulanate NR | | | | 9 | 5 | 55.6 | 1 | 1 | 100 | 5 | 4 | 80 |  | NR |  | 9 | 9 | 100 |
| Carbapenems | 1 | 0 | 0 | 9 | 0 | 0 | 1 | 0 | 0 | 6 | 0 | 0 | 1 | 1 | 100 | 9 | 0 | 0 |
| Cefalexin | | NR |  | 9 | 3 | 33.3 | 0 | 0 |  |  | NR |  |  | NR |  | 1 | 1 | 100 |
| Cefepime | 1 | 0 | 0 | 9 | 4 | 44.4 | 1 | 0 | 0 | 6 | 3 | 50 | 1 | 1 | 100 | 9 | 0 | 0 |
| Ceftazidime | 1 | 0 | 0 | 9 | 4 | 44.4 | 1 | 0 | 0 | 6 | 3 | 50 | 1 | 0 | 0 | 9 | 0 | 0 |
| Ciprofloxacin | 1 | 0 | 0 | 9 | 2 | 22.2 | 1 | 0 | 0 | 6 | 2 | 33.33 | 1 | 1 | 100 | 9 | 0 | 0 |
| Co-trimoxazole | 0 | 0 |  | 4 | 3 | 75.0 | 0 | 0 |  | 3 | 3 | 100 |  | NR |  | 6 | 0 | 0 |
| Gentamicin | 1 | 0 | 0 | 9 | 6 | 66.7 | 1 | 0 | 0 | 6 | 4 | 66.67 | 1 | 1 | 100 | 9 | 0 | 0 |
| Piperacillin-tazobactam | 1 | 0 | 0 |  | NR |  | 1 | 0 | 0 |  | NR |  | 0 | 0 |  | 0 | 0 |  |
| Tobramycin | 1 | 0 | 0 | 9 | 4 | 44.4 | 1 | 0 | 0 | 6 | 3 | 50 | 1 | 1 | 100 | 9 | 0 | 0 |
| Trimethoprim NR | | |  | 5 | 2 | 40 | 1 | 0 | 0 | 3 | 3 | 100 |  | NR |  | 3 | 0 | 0 |

*Not reported because of intrinsic resistance or limited clinical use. Antimicrobials not used for susceptibility tested were omitted from the table. ~3^rd^ generation cephalosporin. N: Number tested; n: Number non-susceptible.

| Supplemental Table 11. Non-susceptibility antibiogram of gram-positive bacteria for site 1 | | | | | | | | | | | | |
| --- | --- | --- | --- | --- | --- | --- | --- | --- | --- | --- | --- | --- |
|  | ***Enterococcus* spp.** | | | **Group A Streptococcus** | | | **Group B Streptococcus** | | | ***S. aureus*** | | |
|  | **N** | **n** | **%** | **N** | **n** | **%** | **N** | **n** | **%** | **N** | **n** | **%** |
| 3GC~ |  | NR* |  | 0 | 0 |  | 1 | 1 | 100 |  | NR |  |
| Aminopenicillin | 8 | 2 | 25.0 |  | NR |  |  | NR |  | 5 | 1 | 20.0 |
| Carbapenems | 9 | 7 | 77.8 |  | NR |  |  | NR |  |  | NR |  |
| Cefoxitin |  | NR |  |  | NR |  |  | NR |  | 2 | 0 | 0 |
| Clindamycin |  | NR |  | 1 | 1 | 100 | 0 | 0 |  | 1 | 0 | 0 |
| Co-trimoxazole |  | NR |  |  | NR |  |  | NR |  | 3 | 0 | 0 |
| Erythromycin |  | NR |  | 1 | 0 | 0 | 1 | 0 | 0 |  | NR |  |
| Penicillin | 7 | 2 | 28.6 | 1 | 1 | 100 | 0 | 0 | 0 | 2 | 1 | 50.0 |
| Teicoplanin | 7 | 0 | 0 |  | NR |  |  | NR |  | 6 | 0 | 0 |
| Vancomycin | 6 | 2 | 33.3 |  | NR |  |  | NR |  | 6 | 0 | 0 |

*Not reported because of intrinsic resistance or limited clinical use. Antimicrobials not used for susceptibility tested were omitted from the table. ~3^rd^ generation cephalosporin.

| Supplemental Table 12. Non-susceptibility antibiogram of gram-positive bacteria for site 2 | | | | | | | | | | | | |
| --- | --- | --- | --- | --- | --- | --- | --- | --- | --- | --- | --- | --- |
|  | ***Enterococcus* spp.** | | | **Group A Streptococcus** | | | **Group B Streptococcus** | | | ***S. aureus*** | | |
|  | **N** | **n** | **(%)** | **N** | **n** | **(%)** | **N** | **n** | **(%)** | **N** | **n** | **(%)** |
| 3GC~ |  | NR* |  | 1 | 0 | 0 | 9 | 0 | 0 |  | NR |  |
| Aminopenicillin | 6 | 0 | 0 |  | NR |  |  | NR |  | 0 | 0 |  |
| Cephazolin |  | NR |  |  | NR |  |  | NR |  | 4 | 0 | 0 |
| Clindamycin |  | NR |  | 1 | 0 | 0 | 9 | 4 | 44.4 | 4 | 0 | 0 |
| Co-trimoxazole |  | NR |  |  | NR |  |  | NR |  | 4 | 0 | 0 |
| Erythromycin |  | NR |  | 1 | 0 | 0 | 9 | 3 | 33.3 |  | NR |  |
| Methicillin |  | NR |  |  | NR |  |  | NR |  | 4 | 0 | 0 |
| Penicillin | 6 | 0 | 0 | 1 | 0 | 0 | 9 | 0 | 0 | 0 | 0 |  |
| Vancomycin | 6 | 0 | 0 |  | NR |  |  | NR |  | 4 | 0 | 0 |

*Not reported because of intrinsic resistance or limited clinical use. Antimicrobials not used for susceptibility tested were omitted from the table. ~3^rd^ generation cephalosporin. N: Number tested; n: Number non-susceptible.

| Supplemental Table 13. Non-susceptibility antibiogram of gram-positive bacteria for site 3 | | | | |
| --- | --- | --- | --- | --- |
|  | ***S. aureus*** | | | |
|  | | **N** | **n** | **%** |
| Aminopenicillin | | 2 | 0 | 0 |
| Cefoxitin | | 2 | 0 | 0 |
| Cephazolin | | 2 | 0 | 0 |
| Clindamycin | | 2 | 0 | 0 |
| Co-trimoxazole | | 2 | 0 | 0 |
| Flu/Dicloxacillin | | 2 | 0 | 0 |
| Methicillin | | 2 | 0 | 0 |
| Penicillin | | 2 | 1 | 50.0 |
| Teicoplanin | | 2 | 0 | 0 |
| Vancomycin | | 2 | 0 | 0 |

| Supplemental Table 14. Non-susceptibility antibiogram of gram-positive bacteria for site 4 | | | | | | | | | |
| --- | --- | --- | --- | --- | --- | --- | --- | --- | --- |
|  | ***Enterococcus* spp.** |  |  | **Group B Streptococcus** |  |  | ***S. aureus*** |  |  |
|  | **N** | **n** | **(%)** | **N** | **n** | **(%)** | **N** | **n** | **(%)** |
| 3GC~ |  | NR* |  | 2 | 0 | 0 |  | NR |  |
| Aminopenicillin | 7 | 4 | 57.1 |  | NR |  | 0 | 0 |  |
| Cefoxitin |  | NR |  |  | NR |  | 5 | 0 | 0 |
| Clindamycin |  | NR |  | 2 | 1 | 50.0 | 5 | 0 | 0 |
| Erythromycin |  | NR |  | 2 | 0 | 0 |  | NR |  |
| Flu/Dicloxacillin |  | NR |  |  | NR |  | 5 | 2 | 40.0 |
| Penicillin | 7 | 4 | 57.1 | 2 | 0 | 0 | 5 | 3 | 60.0 |
| Vancomycin | 7 | 2 | 28.6 |  | NR |  | 5 | 0 | 0 |

*Not reported because of intrinsic resistance or limited clinical use. Antimicrobials not used for susceptibility tested were omitted from the table. ~3^rd^ generation cephalosporin. N: Number tested; n: Number non-susceptible.

| Supplemental Table 15. Non-susceptibility antibiogram of gram-positive bacteria for site 5 | | | | | | | | | | | | |
| --- | --- | --- | --- | --- | --- | --- | --- | --- | --- | --- | --- | --- |
|  |  |  |  |  |  |  |  |  |  |  |  |  |
|  | ***Enterococcus* spp.** |  |  | **Group A Streptococcus** |  |  | **Group B Streptococcus** |  |  | ***S. aureus*** |  |  |
|  | **N** | **n** | **(%)** | **N** | **n** | **(%)** | **N** | **n** | **(%)** | **N** | **n** | **(%)** |
| 3GC~ |  | NR* |  | 0 | 0 |  | 2 | 0 | 0 |  | NR |  |
| Aminopenicillin | 6 | 0 | 0 |  | NR |  |  | NR |  | 0 | 0 |  |
| Cefoxitin |  | NR |  |  | NR |  |  | NR |  | 7 | 3 | 42.9 |
| Clindamycin |  | NR |  | 0 | 0 |  | 10 | 4 | 40.0 | 3 | 1 | 33.3 |
| Co-trimoxazole |  | NR |  |  | NR |  |  | NR |  | 7 | 1 | 14.3 |
| Erythromycin |  | NR |  | 0 | 0 |  | 8 | 3 | 37.5 |  |  |  |
| Penicillin | 6 | 0 | 0 | 1 | 0 | 0 | 12 | 0 | 0 | 2 | 2 | 100 |
| Vancomycin | 0 | 0 | 0 |  | NR |  |  | NR |  | 7 | 0 | 0 |
| *Not reported because of intrinsic resistance or limited clinical use. Antimicrobials not used for susceptibility tested were omitted from the table. ~3^rd^ generation cephalosporin. N: Number tested; n: Number non-susceptible. | | | | | | | | | | | | |

| Supplemental Table 16. Non-susceptibility antibiogram of gram-positive bacteria site 6 | | | | | | | | | | | | |
| --- | --- | --- | --- | --- | --- | --- | --- | --- | --- | --- | --- | --- |
|  | ***Enterococcus* spp.** | |  | **Group B Streptococcus** | | | ***S. aureus*** | |  | ***S. pneumoniae*** | | |
|  | **N** | **n** | **(%)** | **N** | **n** | **(%)** | **N** | **n** | **(%)** | **N** | **n** | **(%)** |
| 3GC~ |  | NR* |  | 5 | 0 | 0 |  | NR |  | 1 | 0 | 0 |
| Aminopenicillin | 1 | 0 | 0 |  | NR |  | 0 | 0 |  | 0 | 0 |  |
| Cefoxitin | | NR |  |  | NR |  | 16 | 1 | 6.3 |  | NR |  |
| Clindamycin | | NR |  | 5 | 3 | 60.0 | 16 | 2 | 12.5 | 0 | 0 |  |
| Co-trimoxazole NR | | |  |  | NR |  | 16 | 1 | 6.3 | 1 | 0 | 0 |
| Erythromycin NR | | |  | 5 | 2 | 40.0 |  | NR |  |  | NR |  |
| Penicillin | 0 | 0 |  | 5 | 0 | 0 | 16 | 15 | 93.8 | 1 | 0 | 0 |
| Vancomycin | 1 | 0 | 0 |  | NR |  | 16 | 0 | 0 | 1 | 0 | 0 |

*Not reported because of intrinsic resistance or limited clinical use. Antimicrobials not used for susceptibility tested were omitted from the table. ~3^rd^ generation cephalosporin. N: Number tested; n: Number non-susceptible.

| Supplemental Table 17. Non-susceptibility antibiogram of gram-positive bacteria site 7 | | | | | | |
| --- | --- | --- | --- | --- | --- | --- |
|  | **Group B Streptococcus** | | | ***S. aureus*** | | |
|  | **N** | **n** | **(%)** | **N** | **n** | **(%)** |
| 3GC~ | 1 | 0 | 0 |  | NR |  |
| Aminopenicillin |  | NR* |  | 0 | 0 |  |
| Cefoxitin |  | NR |  | 7 | 6 | 85.7 |
| Cephazolin |  | NR |  | 6 | 6 | 100 |
| Clindamycin | 2 | 2 | 100 | 7 | 5 | 71.4 |
| Co-trimoxazole |  | NR |  | 6 | 5 | 83.3 |
| Erythromycin | 2 | 2 | 100 |  | NR |  |
| Penicillin | 2 | 0 | 0 | 7 | 6 | 85.7 |
| Teicoplanin |  | NR |  | 7 | 0 | 0 |
| Vancomycin |  | NR |  | 7 | 0 | 0 |

*Not reported because of intrinsic resistance or limited clinical use. Antimicrobials not used for susceptibility tested were omitted from the table. ~3^rd^ generation cephalosporin. N: Number tested; n: Number non-susceptible.

| Supplemental Table 18. Antifungal resistance profiles by species and agent for site 1 | | | | | | | | | | | | | | | |
| --- | --- | --- | --- | --- | --- | --- | --- | --- | --- | --- | --- | --- | --- | --- | --- |
|  | ***C. albicans*** | | | ***C. parapsilosis*** | | | ***C. ciferii*** | | | ***C. guilliermondii*** | | | ***C. pelliculosa*** | | |
|  | **N** | **n** | **%** | **N** | **n** | **%** | **N** | **n** | **%** | **N** | **n** | **%** | **N** | **n** | **%** |
| Amphotericin B | 1 | 0 | 0 | 5 | 0 | 0 | 3 | 1 | 33.3 | 2 | 0 | 0 | 0 | 0 |  |
| Caspofungin | 1 | 0 | 0 | 5 | 0 | 0 | 0 | 0 |  | 2 | 0 | 0 | 0 | 0 |  |
| Fluconazole | 1 | 0 | 0 | 5 | 1 | 20.0 | 0 | 0 |  | 0 | 0 |  | 1 | 0 | 0 |
| Flucytosine | 1 | 0 | 0 | 5 | 0 | 0 | 0 | 0 |  | 2 | 0 | 0 | 0 | 0 |  |
| Micafungin | 1 | 0 | 0 | 5 | 0 | 0 | 0 | 0 |  | 2 | 1 | 50.0 | 0 | 0 |  |
| Voriconazole | 1 | 0 | 0 | 5 | 0 | 0 | 3 | 0 | 0 | 2 | 0 | 0 | 0 | 0 |  |

N: Number tested; n: Number non-susceptible.

| Supplemental Table 19. Antifungal resistance profiles by species and agent for site 2 | | | | | | | | | |
| --- | --- | --- | --- | --- | --- | --- | --- | --- | --- |
|  | ***C. albicans*** | | | ***C. glabrata*** | | | ***C. parapsilosis*** | | |
|  | **N** | **n** | **(%)** | **N** | **n** | **(%)** | **N** | **n** | **(%)** |
| Amphotericin B | 10 | 0 | 0 | 3 | 0 | 0 | 8 | 1 | 12.5 |
| Caspofungin | 10 | 0 | 0 | 3 | 2 | 66.7 | 8 | 0 | 0 |
| Fluconazole | 10 | 0 | 0 | 3 | 0 | 0 | 8 | 2 | 25.0 |
| Micafungin | 10 | 0 | 0 | 3 | 3 | 100 | 8 | 0 | 0 |
| Voriconazole | 10 | 0 | 0 | 3 | 0 | 0 | 8 | 0 | 0 |

N: Number tested; n: Number non-susceptible.

| Supplemental Table 20. Antifungal resistance profiles by species and agent for site 3 | | | | | | | | | | | | |
| --- | --- | --- | --- | --- | --- | --- | --- | --- | --- | --- | --- | --- |
|  | ***C. albicans*** | | | ***C. glabrata*** | | | ***C. parapsilosis*** | | | ***C. tropicalis*** | | |
|  | **N** | **n** | **%** | **N** | **n** | **%** | **N** | **n** | **%** | **N** | **n** | **%** |
| Amphotericin B | 2 | 0 | 0 | 2 | 0 | 0 | 2 | 0 | 0 | 1 | 0 | 0 |
| Caspofungin | 2 | 0 | 0 | 2 | 2 | 100 | 2 | 0 | 0 | 1 | 0 | 0 |
| Fluconazole | 2 | 0 | 0 | 0 | 0 |  | 2 | 0 | 0 | 1 | 0 | 0 |
| Flucytosine | 2 | 0 | 0 | 2 | 0 | 0 | 2 | 0 | 0 | 1 | 0 | 0 |
| Micafungin | 2 | 0 | 0 | 2 | 0 | 0 | 2 | 0 | 0 | 1 | 0 | 0 |
| Voriconazole | 2 | 0 | 0 | 2 | 0 | 0 | 2 | 0 | 0 | 1 | 0 | 0 |

N: Number tested; n: Number non-susceptible.

| Supplemental Table 21. Antifungal resistance profiles by species and agent for site 4 | | | | | | | | | | | | | | | |
| --- | --- | --- | --- | --- | --- | --- | --- | --- | --- | --- | --- | --- | --- | --- | --- |
|  | ***C. albicans*** | | | ***C. glabrata*** | | | ***C. parapsilosis*** | | | ***C. tropicalis*** | | | ***Candida spp.* (other or not speciated)** | | |
|  | **N** | **n** | **(%)** | **N** | **n** | **(%)** | **N** | **n** | **(%)** | **N** | **n** | **(%)** | **N** | **n** | **(%)** |
| Amphotericin B | 3 | 0 | 0 | 1 | 0 | 0 | 16 | 0 | 0 | 10 | 0 | 0 | 4 | 0 | 0 |
| Caspofungin | 3 | 0 | 0 | 1 | 0 | 0 | 16 | 0 | 0 | 10 | 0 | 0 | 4 | 0 | 0 |
| Fluconazole | 3 | 0 | 0 | 1 | 0 | 0 | 16 | 0 | 0 | 10 | 0 | 0 | 4 | 0 | 0 |
| Flucytosine | 3 | 0 | 0 | 1 | 0 | 0 | 16 | 0 | 0 | 10 | 0 | 0 | 4 | 0 | 0 |
| Micafungin | 3 | 0 | 0 | 1 | 0 | 0 | 16 | 0 | 0 | 10 | 0 | 0 | 4 | 0 | 0 |
| Voriconazole | 3 | 0 | 0 | 1 | 0 | 0 | 16 | 0 | 0 | 10 | 0 | 0 | 4 | 0 | 0 |

N: Number tested; n: Number non-susceptible.

| Supplemental Table 22. Antifungal resistance profiles by species and agent for site 5 | | | | | | |
| --- | --- | --- | --- | --- | --- | --- |
|  | ***C. albicans*** | | | ***Candida spp.* (other or not speciated)** | | |
|  | **N** | **n** | **(%)** | **N** | **n** | **(%)** |
| Fluconazole | 1 | 0 | 0 | 1 | 0 | 0 |

N: Number tested; n: Number non-susceptible.

**Note:** No fungal isolates available for site 6.

| Supplemental Table 23. Antifungal resistance profiles by species and agent for site 7 | | | | | | |
| --- | --- | --- | --- | --- | --- | --- |
|  | ***C. albicans*** | | | ***C. parapsilosis*** | | |
|  | **N** | **n** | **(%)** | **N** | **n** | **(%)** |
| Amphotericin B | 1 | 0 | 0 | 8 | 0 | 0 |
| Caspofungin | 1 | 0 | 0 | 8 | 0 | 0 |
| Fluconazole | 1 | 0 | 0 | 8 | 0 | 0 |
| Flucytosine | 1 | 0 | 0 | 8 | 0 | 0 |
| Micafungin | 1 | 0 | 0 | 8 | 0 | 0 |
| Voriconazole | 1 | 0 | 0 | 8 | 0 | 0 |

N: Number tested; n: Number non-susceptible.

**Note:** No fungal isolates identified at sites 8, 9 or10.

# Supplemental Figure 2. Unadjusted antimicrobial non-susceptibility for Enterobacterales by site, pathogen and antimicrobial

# Supplemental Figure 3. Unadjusted antimicrobial non-susceptibility for Gram-negative non-fermenters by site, pathogen and antimicrobial

# Supplemental Figure 4. Unadjusted antimicrobial non-susceptibility for gram-positive bacteria by site, pathogen and antimicrobial
